# CSAKD: Determining Absolute Ligand Affinities From ^19^F NMR Chemical Shift Anisotropy

**DOI:** 10.1002/anie.6036832

**Published:** 2026-06-12

**Authors:** Simon H. Rüdisser, Gabriela Stadler, Alvar D. Gossert

**Affiliations:** ^1^ Department of Biology ETH Zürich Zürich Switzerland

**Keywords:** affinity determination, chemical shielding anisotropy, fragment‐based drug discovery, machine learning, 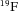
 NMR

## Abstract

Small‐molecule drug discovery typically begins with the screening of compound libraries to identify initial hits, which are subsequently optimized into lead compounds and, ultimately, drug candidates. Diverse screening methodologies are employed, including DNA‐encoded library technology, high‐throughput screening, and fragment‐based drug discovery (FBDD). Among these, FBDD is particularly powerful when integrated with structure‐guided drug design and biophysical affinity measurements. However, accurately quantifying the weak binding affinities of fragments remains a significant challenge. To address this, we introduce chemical shift anisotropy $K_{D}$ (CSAKD), a novel method for determining absolute fragment affinities using 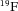 NMR relaxation. The CSAKD approach eliminates the need for titration experiments and isotopic labeling. Furthermore, we complement this method with a machine learning model for the rapid and accurate prediction of 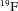 chemical shielding tensors. In summary, CSAKD allows fast and efficient affinity determination which seamlessly integrates into FBDD by 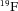 NMR.

## Graphical abstract

Affinity determination is crucial in drug discovery, yet remains difficult for weakly binding fragments. We introduce chemical shift anisotropy $K_{D}$ (CSAKD) by  NMR relaxation experiments, a titration‐free method that requires no isotopic labeling. We also present a machine learning model for predicting  CSA tensors to make CSAKD easily accessible. CSAKD is accurate and requires only a single sample at low μM receptor concentration.

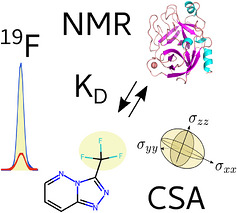

## 1 Introduction

The search of chemical starting points in target‐based drug discovery employs diverse screening paradigms. While high‐throughput screening (HTS) assesses vast libraries of lead‐like molecules [1], fragment‐based drug discovery (FBDD) offers a complementary strategy by interrogating smaller, simpler compounds with high ligand efficiency [2, 3]. These fragments provide superior coverage of chemical space and are optimized into potent leads through structure‐guided design [4]. As weak fragment binding necessitates sensitive, label‐free detection methods, biophysical techniques like surface plasmon resonance, NMR, and x‐ray crystallography are paramount, being less susceptible to the assay interference common at high screening concentrations [5, 6, 7, 8].

Among these, 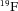 NMR has emerged as a particularly powerful technique for fragment screening due to its high sensitivity and experimental simplicity [9]. The development of libraries featuring diverse local fluorine environments (LEF), combined with ligand‐observed 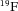 NMR experiments, provides a robust methodology for initial hit identification [10]. However, the transition from qualitative binding detection to precise, quantitative affinity determination for these weak binders remains a challenge. Current NMR methods often rely on titration‐based approaches, which are material‐intensive, require isotopic labeling and are time consuming [5, 6, 7, 8]. It is this critical gap that our novel method, chemical shift anisotropy $K_{D}$ (CSAKD), is designed to address. Unlike established ligand‐observed methods, such as 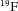 reporter assays [11], WaterLOGSY [12], or STD [13] which require data collection at multiple ligand to receptor ratios, CSAKD requires only a single sample. This new approach enables accurate $K_{D}$ determination without isotopic labeling and at an about 10 times lower receptor concentrations than target‐observed NMR titration experiments [14].

In order to determine the relative affinities of 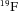 screening hits, we have recently introduced the CSAR (chemical shift‐anisotropy‐based affinity ranking) method [15]. Affinity ranking is essential to prioritize hits for hit‐to‐lead progression. CSAR allows the ranking of ligands based on 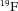 CSA relaxation data without the need for titration experiments [15]. Briefly, the CSAR method consists of three steps: 1) The CSA contribution to $R_{2}$, $R_{\text{2,CSA}}$, of 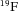 ligands is measured by separating it from the exchange and dipole–dipole $R_{2}$ relaxation. 2) The theoretical $R_{\text{2,CSA}}$ of the free ligand is calculated using density functional theory (DFT) methods. 3) The ratio of measured to calculated $R_{\text{2,CSA}}$ values is proportional to the fraction of bound ligand and therefore provides an accurate affinity ranking for ligands binding to the same receptor. Affinity ranking using CSAR is invaluable for prioritizing hits identified through NMR screening. Relative affinity data derived from CSAR enables the generation of structure–activity relationships (SAR), which are crucial for guiding medicinal chemistry efforts, particularly when structural details of the target–ligand complex are unavailable. However, the original CSAR approach cannot determine absolute binding affinities, typically expressed by the dissociation constant $K_{D}$ [15]. $K_{D}$ values are necessary for comparing affinities across biophysical methods such as surface plasmon resonance (SPR), isothermal titration calorimetry (ITC) [16], or $IC_{50}$ values from enzymatic assays [17]. $K_{D}$ values provide critical input for computational models predicting binding free energy, enhancing our understanding of the physical mechanisms underlying target–ligand interactions [18].

CSAKD extends the established CSAR methodology [15] by incorporating accurate calculations of the $R_{\text{2,CSA}}$ relaxation rate of the ligand in the bound state. These calculations explicitly account for 1) anisotropic rotational diffusion by hydrodynamic calculations [19], 2) internal dynamics using the model free approach [20, 21], and 3) the 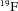 CSA tensor of the ligand. We demonstrate experimentally that CSAKD yields accurate $K_{D}$ values for four distinct protein targets. Furthermore, theoretical analysis indicates that the unknown internal dynamics and binding mode of the bound ligand do not compromise the accuracy of the CSAKD method. Consequently, CSAKD is shown to be broadly applicable to a diverse range of targets.

The calculation of $R_{\text{2,CSA}}$ for CSAKD requires the knowledge of the CSA tensor for the ligand. In this work, we present a novel method to accurately predict the CSA of 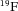 from molecular fingerprints by using machine learning methods. The prediction based on molecular fingerprints is about 7000 times faster than much more resource‐intensive DFT calculations. We provide the machine learning‐predicted CSA values for the entire set of 1207 molecules comprising the OpenFL600 fragment screening library, which is described in Rüdisser et al., 2026. With these improvements, CSAKD becomes accessible without the need of extensive computing infrastructure.

**FIGURE 1:**
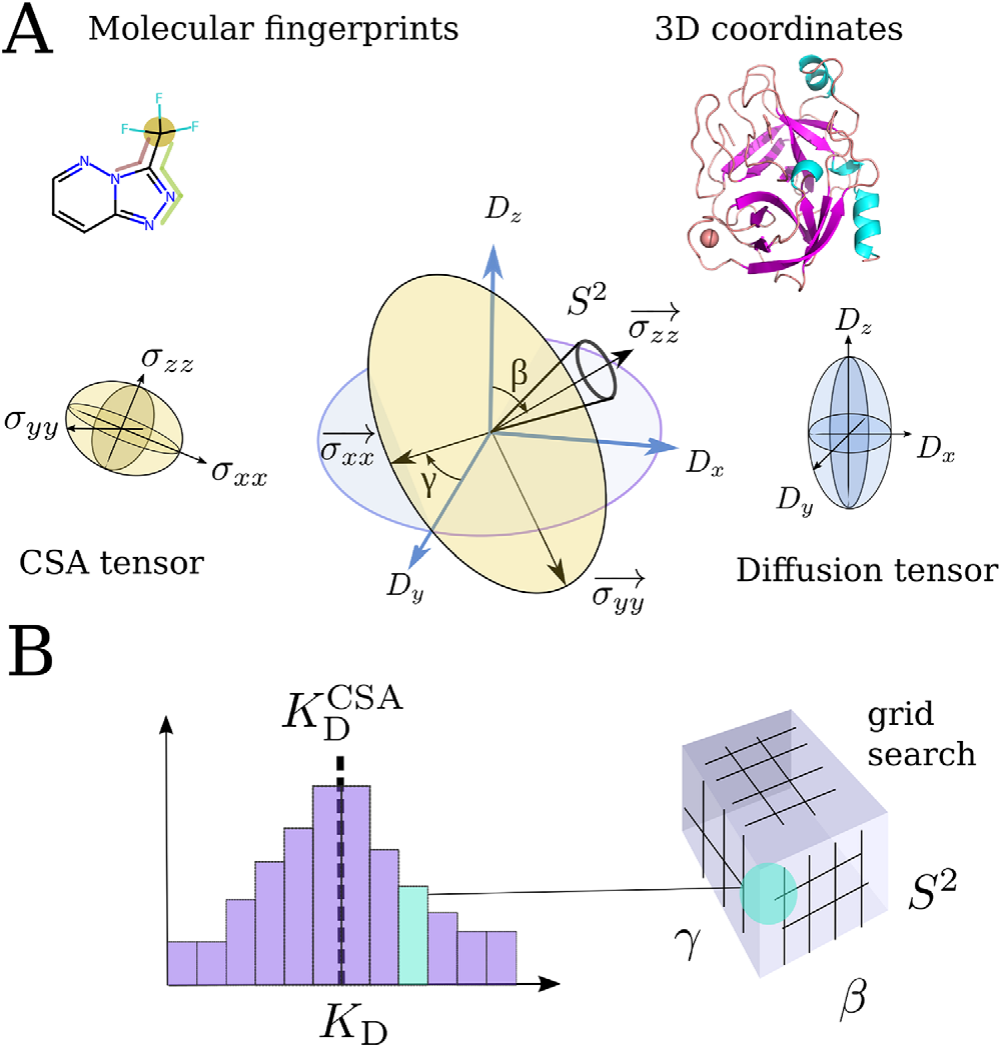
(A) Illustration of $K_{D}$ determination by the CSAKD approach. First, the CSA of 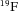 for the ligand is calculated from local environment of fluorine (LEF) molecular fingerprints [10] which are subsequently used as input for random forest [22] regression. On the side of the receptors, the diffusion tensor of the receptors is computed via hydrodynamic simulations using HYDRONMR [19]. (B) The ligand affinity is calculated by grid‐searching the order parameter $S^{2}$, and the Euler angles β and γ. The $K_{D}$ of the ligand is within the resulting distribution of possible $K_{D}$ values.

Our workflow streamlines affinity determination by 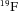 NMR in early drug discovery by efficient CSA and diffusion tensor calculation, as well as $R_{\text{2,CSA}}$ measurements. An overview of this process is provided in Figure 1.

## 2 Results and Discussion

The determination of $K_{D}$ values using the newly introduced CSAKD (chemical shift anisotropy $K_{D}$) method will be described in the following sections. The CSAKD method relies on experimentally determined 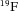 $R_{\text{2,CSA}}$ rates of the ligand in the absence and presence of the receptor, as well as theoretical $R_{\text{2,CSA}}$ rates of the ligand in the bound state. Experimental $R_{\text{2,CSA}}$ values are obtained using the approach described by Rüdisser et al. [15], while theoretical $R_{\text{2,CSA}}$ values are calculated from 1) 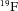 CSA tensors and 2) the diffusion tensor of the receptor. Thus, first the theory will be introduced, and then two sections are dedicated to 1) efficient calculation of 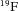 CSA tensors and 2) the rotational correlation time $\tau_{c}$ of the bound ligand, taking into account internal motions of the ligand in the binding pocket and deviations of the receptor from spherical shape.

### 2.1 Theory

The goal of this theory section is to derive an expression that describes the binding dissociation equilibrium constant, $K_{D}$, in terms of parameters, which can be either measured experimentally or calculated. Here, the fraction of bound ligand, $p_{b}$, will be used to compute the $K_{D}$‐value according to equation 1 where ${\left[P\right]}_{\text{tot}}$ and ${\left[L\right]}_{\text{tot}}$ are the total receptor and ligand concentrations, respectively.

(1) $$\begin{array}{c} K_{D}=\frac{\left({\left[P\right]}_{\text{tot}}-p_{b}{\left[L\right]}_{\text{tot}}\right)\left(1-p_{b}\right)}{p_{b}} \end{array}$$

The fraction of bound ligand, $p_{b}$, will then be derived from the observed relaxation rate of the ligand, which is a population weighted average of the free and bound relaxation rates—where $p_{b}$ is directly representing the bound population. Equations which are relevant for the description of relaxation processes in 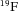 NMR are presented in the following section.

Ligand‐observed NMR screening methods detect weakly binding molecules that are in fast or intermediate exchange on the NMR timescale ($k_{\text{ex}}\geq 1000s^{-1}$). Here, $k_{\text{ex}}=k_{\text{on}}{\left[P\right]}_{\text{free}}+k_{\text{off}}$, with ${\left[P\right]}_{\text{free}}$ representing the concentration of the free receptor. In this time regime, the effective transverse relaxation rate ($R_{2}^{\text{eff}}$) is a population‐weighted average of $R_{2}$ in the free and bound states, indicated by $R_{2,f}$ and $R_{2,b}$, respectively.

(2) $$R_{2}^{\text{eff}}=p_{f}R_{2,f}+p_{b}R_{2,b}+R_{\text{ex}}$$

The fractions of the free and bound ligand are $p_{f}$ and $p_{b}$, respectively, with $p_{f}=1-p_{b}$. An additional exchange term, $R_{\text{ex}}$, accounts for line broadening due to chemical exchange between the free and bound states (Equation 2). $R_{\text{ex}}$ depends on the difference in the resonance frequencies between the free and bound states ($\Delta \omega =\omega_{f}-\omega_{b}$) and the exchange rate between the states, $k_{\text{ex}}$ (Equation 3).

(3) $$R_{\text{ex}}=\frac{p_{f}p_{b}{\left(\omega_{f}-\omega_{b}\right)}^{2}}{k_{\text{ex}}}$$

The two contributions to $R_{2}$ are $R_{2,\text{DD}}$ and $R_{2,\text{CSA}}$ which are the dipolar relaxation and the relaxation from CSA, respectively (Equation 4).

(4) $$R_{2}=R_{2,\text{DD}}+R_{2,\text{CSA}}$$

The dipolar relaxation of spin‐1/2 nuclei depends on the distance (r) between the two nuclei involved (Equation 5). $\gamma_{F}$ and $\gamma_{H}$ are the gyromagnetic ratios of the nuclei, which in this example are 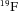 and 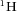. ℏ is the reduced Planck constant (h/(2π)) and $\mu_{0}$ is the magnetic permeability in the vacuum. J(ω) are the spectral densities at the corresponding frequencies.

(5) $$\begin{array}{c} R_{2,\text{DD}}=\frac{1}{20}\hbar^{2}\mu_{0}^{2}\gamma_{F}^{2}\gamma_{H}^{2}\sum_{i}\frac{1}{r_{\left(F,H_{i}\right)}^{6}}[4J(0)+3J(\omega_{F})\\ +6J(\omega_{H})+J(\omega_{F}-\omega_{H})+6J(\omega_{F}+\omega_{H})] \end{array}$$

$R_{2,\text{DD}}$ is, to a first approximation, independent of the external magnetic field. The $R_{2,\text{CSA}}$ term (Equation 6) exhibits a quadratic $B_{0}$‐field dependence and depends on the shape of the chemical shielding tensor.

(6) $$R_{2,\text{CSA}}=\frac{1}{20}\gamma_{F}^{2}B_{0}^{2}\delta_{\sigma }^{2}\left(1+\frac{\eta^{2}}{3}\right)\left[4J\left(0\right)+3J\left(\omega_{F}\right)\right]$$

The shape of the shielding tensor is described in NMR literature by two equivalent expressions: either by the overall anisotropy, $\Delta \sigma =\sigma_{\text{zz}}-\left(\sigma_{\text{xx}}+\sigma_{\text{yy}}\right)/2$, or by the combined variables of reduced anisotropy, $\delta_{\sigma }=\sigma_{\text{zz}}-\sigma_{\text{iso}}$, and axial asymmetry [23, 24], $\eta =\left(\sigma_{\text{yy}}-\sigma_{\text{xx}}\right)/\delta_{\sigma }$. $\sigma_{\text{xx}}$, $\sigma_{\text{yy,}}$ and $\sigma_{\text{zz}}$ denote the magnitude of the shielding tensor in the principal axis frame along the corresponding axis. Note that in Equation 6, all variables on the left hand side are known and the reduced anisotropy can be calculated.

Later in this work, we will introduce a machine‐learning model to predict the CSA tensor from molecular fingerprints. In order to simplify the model for the predictions of the CSA tensor, we combine the anisotropy and asymmetry into one single parameter, the effective anisotropy, $\delta_{\sigma }^{\text{eff}}$, which is defined in Equation 7. $\delta_{\sigma }^{\text{eff}}$ is equal to $\delta_{\sigma }$ for a symmetric tensor. $\delta_{\sigma }^{\text{eff}}$ can be directly used to calculate $R_{\text{2,CSA}}$ according to Equation 6.

(7) $$\begin{array}{c} \delta_{\sigma }^{\text{eff}}=\delta_{\sigma }\sqrt{1+\frac{\eta^{2}}{3}} \end{array}$$

The spectral densities in Equations 5 and 6, J(ω), can also be calculated. They depend on the frequency ω, the tumbling rate of the molecule and internal mobility. Internal mobility is commonly described using the model‐free approach [20, 21], where the spectral density is expressed by Equation 8.

(8) $$J\left(\omega \right)=2\left[\frac{S^{2}\tau_{c}}{1+{\left(\omega \tau_{c}\right)}^{2}}+\frac{\left(1-S^{2}\right)\tau_{i}}{1+{\left(\omega \tau_{i}\right)}^{2}}\right]$$

In this approach, $S^{2}$ represents the generalized order parameter, which quantifies the amplitude of internal motion on a cone, and $\tau_{i}$ denotes the internal correlation time [20, 21]. For spherical molecules undergoing isotropic rotational diffusion, a single rotational correlation time, $\tau_{c}$, is sufficient to describe J(ω). Note that $\tau_{i}\ll \tau_{c}$.

For non‐spherical molecules undergoing anisotropic rotational diffusion, the principal components of the diffusion tensor, $D_{x}$, $D_{y}$, and $D_{z}$, are required to calculate the spectral densities. The anisotropy (ζ) and and rhombicity ($\eta_{D}$) of the diffusion tensor are $\zeta =2D_{z}/\left(D_{x}+D_{y}\right)$ and $\eta_{D}=1.5\left(D_{y}-D_{x}\right)/\left[D_{z}-0.5\left(D_{x}+D_{y}\right)\right]$ [25]. Note that we use the symbol $\eta_{D}$ for the rhombicity of the diffusion tensor in order to discriminate it from η, which is the asymmetry of the CSA tensor. Additionally, the spectral densities depend on the relative orientation of the diffusion tensor with respect to the CSA tensor. In the case of axially symmetric diffusion ($\eta_{D}=0$), the Euler angles β and γ describe the relative orientation of the two tensors [26]. Detailed expressions for the spectral densities are provided in the Supporting Information (Equations S6–S13).

Under strong spin‐lock conditions, the exchange term $R_{\text{ex}}$ in Equation 2 can be suppressed [15]. The remaining contributions from $R_{2,\text{DD}}$ and $R_{2,\text{CSA}}$ can be separated by measuring at different external magnetic fields ($B_{0_{1}}$ and $B_{0_{2}}$).

(9) $$\begin{array}{c} \Delta R_{2}^{\text{SL}}=p_{f}R_{2,f,B_{0_{2}}}^{\text{SL}}+p_{b}\left(R_{2,\text{CSA,b},B_{0_{2}}}+R_{2,\text{DD,b},B_{0_{2}}}\right)\\ -p_{f}R_{2,f,B_{0_{1}}}^{\text{SL}}-p_{b}\left(R_{2,\text{CSA,b},B_{0_{1}}}+R_{2,\text{DD,b},B_{0_{1}}}\right) \end{array}$$

And conversely, $p_{b}$ can be expressed as follows:

(10) $$\begin{array}{c} p_{b}=\frac{\Delta R_{2}^{\text{SL}}-\Delta R_{2,f}^{\text{SL}}}{\Delta R_{2,\text{CSA},b}+\Delta R_{2,\text{DD},b}-\Delta R_{2,f}^{\text{SL}}} \end{array}$$

The terms $\Delta R_{2,\text{CSA},b}$ and $\Delta R_{2,\text{DD},b}$ denote the differences in CSA and DD relaxation rates for the bound ligand between two magnetic fields, $B_{0_{1}}$ and $B_{0_{2}}$. $\Delta R_{2,f}^{\text{SL}}$ represents the total difference in relaxation rates ($R_{2}^{\text{SL}}$) under spin‐lock conditions for the free ligand between $B_{0_{1}}$ and $B_{0_{2}}$. For the free ligand, the individual CSA and DD contributions to $\Delta R_{2,f}^{\text{SL}}$ are not resolved, as this term serves only to correct for the relaxation of the free ligand in samples containing both free and bound states. For the bound ligand, however, separating the CSA and DD components is essential for calculating the affinity of the ligand with the CSAKD approach.

This separation is possible because the CSA contribution ($\Delta R_{2,\text{CSA},b}$) scales with the square of the magnetic field strength, whereas the DD contribution ($\Delta R_{2,\text{DD},b}$) is, to a first approximation, field‐independent (Equation 5). Consequently, subtracting the relaxation rate at the first field ($R_{2,B_{0_{1}}}^{\text{SL}}$) from that at the second field ($R_{2,B_{0_{2}}}^{\text{SL}}$) causes the dipolar contributions to cancel, thereby isolating the CSA effect [15] (Equation 11).

(11) $$\begin{array}{cc} p_{b} & =\frac{\Delta R_{2}^{\text{SL}}-\Delta R_{2,f}^{\text{SL}}}{\Delta R_{2,\text{CSA,b}}-\Delta R_{2,f}^{\text{SL}}}\\ & =\frac{\Delta R_{2,\text{obs}}^{\text{SL}}-\Delta R_{2,\text{f, obs}}^{\text{SL}}}{\Delta R_{2,\text{CSA,b,calc}}-\Delta R_{2,\text{f, obs}}^{\text{SL}}} \end{array}$$

Due to the small CSA contribution to $R_{2}$ relaxation in rapidly tumbling small molecules, $\Delta R_{2,f}$ exhibits a weaker field dependence than $\Delta R_{2,\text{CSA},b}$.

In summary, by comparing the measured $R_{2}^{\text{SL}}$, which represents a population‐weighted average of free and bound ligand, to the calculated value of $R_{2,b}$, that is, if 100% were bound, allows one to derive the fraction of bound ligand $p_{b}$. To this end, $R_{2}$ must be simplified by removing $R_{2,\text{DD}}$ and suppressing $R_{\text{ex}}$ such that only contributions that can be calculated remain, that is, $R_{\text{2,CSA}}$.

### 2.2 An Accurate and Fast Machine Learning Method Based on One‐Dimensional Molecular Fingerprints to Predict the CSA of 

The CSA contribution to $R_{2}$ relaxation, $R_{\text{2,CSA}}$, is determined by the shape of the CSA tensor. The CSA tensor can be accurately calculated using DFT methods, for instance with the computer program *Gaussian09* [15, 27, 28]. Calculating the CSA tensor with DFT methods, however, requires computational resources which are often not available. Therefore, we introduce a computationally inexpensive and accurate approach for predicting the CSA from molecular fingerprints with random‐forest regression. In a first step, the molecular environment of the fluorine atom is described by paths of connected atoms which are originating at the fluorine atom for CF and at the carbon atom for ${\mathrm{CF}}_{3}$. The nodes of a path correspond to atoms and are classified according to the atom type. The paths are then encoded as a one‐dimensional fingerprint. These fingerprints are particularly suitable to describe the local environment of fluorine (LEF) and have been applied to predict the chemical shift of 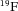 [10, 29].

**FIGURE 2:**
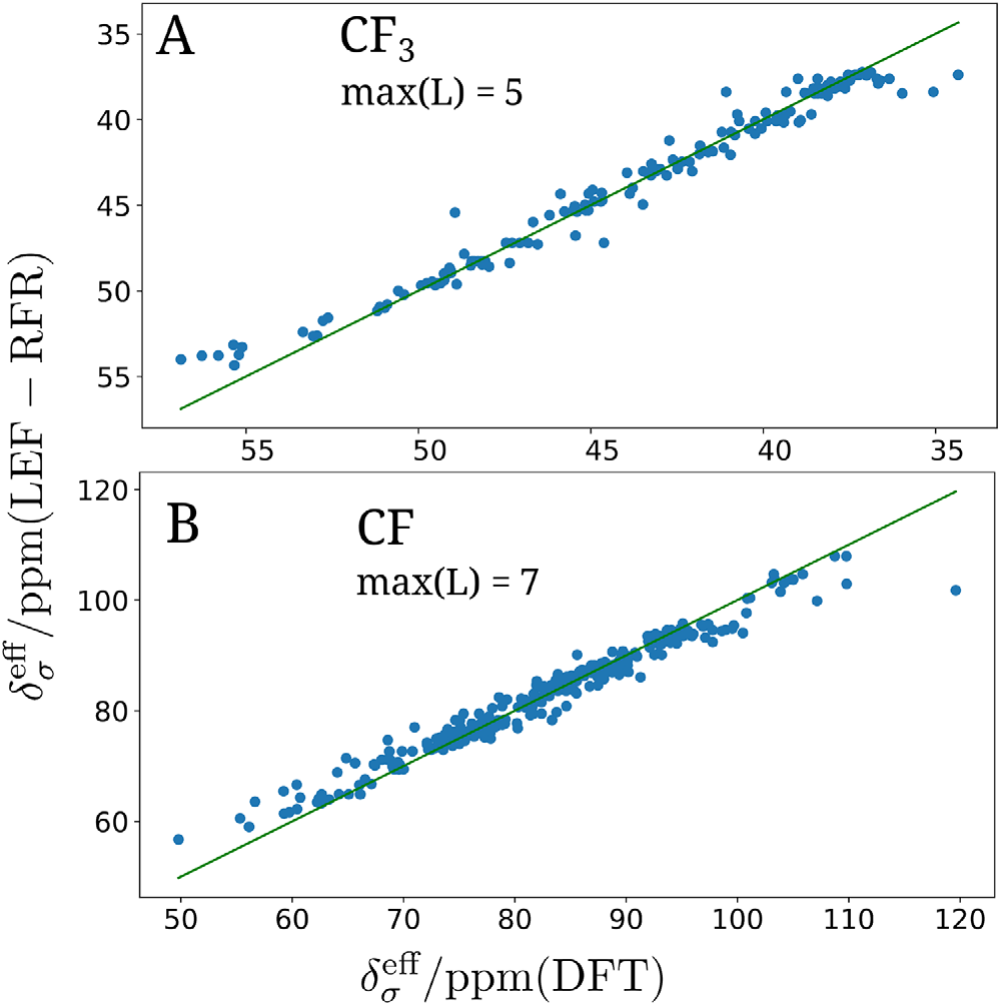
LEF ‐ random forest regression (RFR) predicted versus DFT calculated $\delta_{\sigma }^{\text{eff}}$ values are show for (A) 181 ${\mathrm{CF}}_{3}$ and (B) 326 CF molecules. These molecules are a subset of the OpenFL600 fragment‐screening library which is described in Rüdisser et al., 2026. The maximum path length for the LEF fingerprints, max(L), determines the accuracy of the prediction by random forest regression. The max(L) values which result in the minimum RMSE are 7 for CF (RMSE = 2.26 ppm) and 5 for ${\mathrm{CF}}_{3}$ (RMSE = 0.89 ppm).

A random‐forest regressor [30] has been trained on LEF fingerprints and $\delta_{\sigma }^{\text{eff}}$ which has been calculated using DFT methods. The maximal path length, max(L), for the calculation of LEF fingerprints determines the number of bonds included in LEF fingerprints. High max(L) values allow to include effects of atoms distant from the fluorine atom. We have investigated the influence of the maximal path length, max(L), on the predictor performance. Furthermore, to determine the optimal parameters for the random‐forest regression, a grid search and 3‐fold cross‐validation was conducted. The optimal max(L) yielding the lowest root mean square error (RMSE), along with the random forest parameters, was determined separately for CF and ${\mathrm{CF}}_{3}$ molecules (see Supporting Information). CF and ${\mathrm{CF}}_{3}$ result in different max(L), since the descriptor begins counting from the bond with the carbon atom in ${\mathrm{CF}}_{3}$, while for CF, counting starts directly at the fluorine atom. Additionally, we hypothesize that the CSA of CF, described by the parameters $\delta_{\sigma }$ and η, exhibits greater sensitivity to distant substituents compared to the symmetric CSA of ${\mathrm{CF}}_{3}$, where η = 0. This is also reflected in the slightly elevated RMSE for CF compared to ${\mathrm{CF}}_{3}$. Figure 2 shows the regression which was obtained using the optimized parameters. The achieved RMSE for $\delta_{\sigma }^{\text{eff}}$ is around 2 ppm, which results in an error of less than 5% for the $R_{\text{2,CSA}}$ relaxation rate. The time savings by using our newly introduced fingerprint‐based CSA predictor are substantial. The speedup is more than 7000 times compared to DFT calculations as detailed in the Supporting Information.

### 2.3 Calculation of the Diffusion Tensor of Receptors

The relationship between $R_{\text{2,CSA}}$ and the rotational correlation time $\tau_{c}$ is expressed by Equation 6. For isotropic tumbling (*e.g*., spherical molecules, $D_{x}=D_{y}=D_{z}$), a single $\tau_{c}$ value is sufficient to describe the tumbling rate. However, in the case of anisotropic tumbling, all three diffusion tensor components, $D_{x},D_{y},D_{z}$, must be known to compute $R_{\text{2,CSA}}$ from spectral densities.

Typically, the diffusion tensor and the rotational correlation time for proteins is derived from ^15^N‐{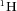} NOE and ^15^N T_1_/T_2_ or ^13^C‐{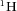} NOE and ^13^C T_1_/T_2_ relaxation data [25, 31]. However, this approach necessitates isotopic enrichment of the protein with ^15^N or ^13^C, which is often impractical or unfeasible. For the CSAKD method, we predict the diffusion tensor from atomic‐level structures using hydrodynamic calculations. Specifically, the HYDRONMR software [19], based on bead‐model methodology, provides accurate diffusion tensor values for sufficiently rigid proteins.

### 2.4 $R_{\text{2,CSA}}$ is Dependent on the Internal Dynamics of the Ligand and the Binding Pose

Molecular motions of the ligand that are significantly faster than overall rotational tumbling, characterized by an internal correlation time ($\tau_{i}\ll \tau_{c}$), lead to a reduction in $R_{\text{2,CSA}}$. The rapid dynamics of a bound ligand arise from intramolecular flexibility [32] (e.g., rotations around dihedral angles), mobility within the binding pocket, and fast exchange between multiple binding poses, all of which contributes to the conformational entropy of binding [33, 34]. The order parameter $S^{2}$ is commonly used to quantify local dynamics in proteins and nucleic acids [20, 21]. The values of $S^{2}$ for protein backbone and side chains vary due to steric interactions that restrict molecular motion and the inherent flexibility of specific amino acids [35, 36]. For the relatively rigid protein backbone, $S^{2}$ typically averages around 0.85. In contrast, for the more mobile side‐chain methyl groups, $S^{2}$ decreases as the side chain length increases [36]. Among these methyl groups, alanine exhibits the least mobility, with $S^{2}$ values ranging from 0.6–1.0 and a distribution maximum at 0.85. To the best of our knowledge, order parameters for ligands in the bound state have been determined in only a few cases, which are discussed below. $S^{2}$ values have been measured for p‐Gly_n_‐substituted benzenesulfonamide inhibitors (*n* = 1⋯6) bound to bovine carbonic anhydrase II to estimate residual ligand entropy [37]. In another application, the order parameter $S^{2}$ of AST‐487, a ${\mathrm{CF}}_{3}$‐containing kinase inhibitor, was determined in the p38α‐bound state using forbidden coherence transfer measurements. The resulting $S^{2}$ = 0.64 ± 0.07 indicates that the ${\mathrm{CF}}_{3}$ group, directly attached to a phenyl ring, retains significant mobility in the bound state [38]. A similar approach applied to the CH_3_ groups of a peptidic inhibitor bound to myocyte‐enhancer factor 2A (MEF2A) yielded $S^{2}$ values ranging from approximately 0.45– 0.6 for Val $\gamma_{1}$ and $\gamma_{2}$ CH_3_, reflecting the surface complementarity between the ligand and receptor [39]. Based on the reported $S^{2}$ values for methyl side chains and receptor‐bound ligands, we estimate $S^{2}$ values within the interval $S^{2}\in \left[0.5,0.85\right]$ for a bound ligand. Furthermore, internal motions occurring on timescales faster than 100 ps, as commonly observed for local protein side‐chain dynamics [36], have negligible effect on $R_{\text{2,CSA}}$ (see Supporting Information, Figure S5). The $R_{\text{2,CSA}}$ of the bound ligand also depends on the orientation of the CSA tensor relative to the diffusion tensor. The relative orientation is defined by the Euler angles β and γ for an axially symmetric diffusion tensor (see Supporting Information, Equations S6– S13).

To account for these parameters, $K_{D}^{\text{CSA}}$ values are computed over a range of values: $S^{2}\in \left[0.5,0.85\right]$, β∈[0,π], and γ∈[0,π/2]. These parameters were uniformly sampled on a 20×20×20 grid. Uniform sampling of the spherical surface for the Euler angles β and γ was achieved by setting β=arccos(2u−1) where u∈[0,1]. The reported $K_{D}^{\text{CSA}}$ is the mean of the 8000 computed values, along with the standard error. Thus, the standard error in $K_{D}^{\text{CSA}}$ arises from the unknown contribution of fast internal dynamics ($S^{2}$) as well as the unknown relative orientation of the diffusion and CSA tensors (β and γ).

### 2.5 The Accuracy of $K_{D}^{\text{CSA}}$ is not Compromised by the Unknown Parameters $S^{2}$, β, and γ

**FIGURE 3:**
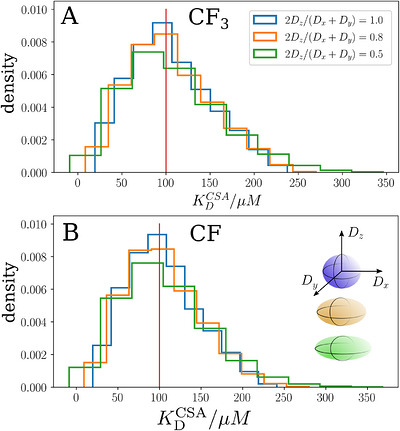
$K_{D}^{\text{CSA}}$ values were calculated for theoretical $\Delta R_{\text{2,CSA}}$ to evaluate the influence of the unknown parameters $S^{2},\beta$, and γ. $\Delta R_{\text{2,CSA}}$ rates were calculated using the following parameters: $K_{D}$ = 100 µM, c(ligand) = 100 µM, c(receptor) = 10 µM, $\Delta R_{\text{2,CSA,f}}$ = 0.3 $s^{-1}$, $B_{0_{1}}$ = 16.4 T, $B_{0_{2}}$ = 11.75 T, $\tau_{i}$ = 1 ps, (A) for ${\mathrm{CF}}_{3}$ $\delta_{\sigma }$ = 43.52 ppm and η = 0 ppm and (B) for CF: $\delta_{\sigma }$ = 69.6 and η = 0.998. The sum $D_{x}+D_{y}+D_{z}$ has been kept constant to 3 × 0.25 10^7^ $s^{-1}$ for the various anisotropies of the diffusion tensor. The $K_{D}^{\text{CSA}}$ values for different diffusion tensor anisotropies, ζ=1.0,0.8,0.5, are provided below: CF_3_: $K_{D}^{\text{CSA}}$ = 101 ± 42, 101 ± 44, and 103 ± 52 µM; CF: $K_{D}^{\text{CSA}}$ = 104 ± 43, 104 ± 44, 106 ± 53 µM. The three different diffusion tensor anisotropies, ζ, are depicted in the lower panel. Note that for ζ<1, the shape of the receptor resembles a prolate spheroid, whereas the diffusion tensor corresponds to an oblate spheroid, as shown in the inset of (B).

To evaluate the influence of the unknown parameters $S^{2},\beta$, and γ on $K_{D}^{\text{CSA}}$, we performed simulations for a ligand with $K_{D}$ = 100 µM, at a ligand concentration of 100 µM and a receptor concentration of 10 µM. The fraction of bound ligand, $p_{b}$, was calculated according to the single binding site model. $\Delta R_{\text{2,CSA}}$ was calculated for three different anisotropies of the diffusion tensor (ζ = 1.0, 0.8, 0.5), with parameter ranges $S^{2}\in \left[0.5,0.85\right]$, β∈[0,π], and γ∈[0,π/2], sampled on a 4×4×4 grid. The CSA parameters $\delta_{\sigma }$ and η for CF and CF_3_ are typical values for the OpenFL600 screening libraries which are described in Rüdisser et al., 2026. The simulation results are presented in Figure 3 for both CF and CF_3_ groups. The $K_{D}^{\text{CSA}}$ distribution peaks at 100 µM, with its width increasing modestly as the diffusion tensor's anisotropy increases. Although the distribution is slightly skewed toward higher $K_{D}$ values, the standard deviation provides a reasonable first‐order estimate of the uncertainty in $K_{D}^{\text{CSA}}$. In summary, the uncertainty in $K_{D}^{\text{CSA}}$ is approximately 50% of the $K_{D}$, which falls within the typical uncertainty range of other $K_{D}$ determination methods. Notably, the uncertainty in $K_{D}^{\text{CSA}}$ is similar for CF and CF_3_ groups. In conclusion, our theoretical investigations demonstrate that affinity determination by CSAKD yields accurate $K_{D}^{\text{CSA}}$ values with a standard error of about 50% for rotational diffusion anisotropies ζ up to 0.5.

### 2.6 CSAKD for PPAT, M^pro^, Abl1, and Trypsin

To evaluate the CSAKD method, we determined $K_{D}^{\text{CSA}}$ values for a series of ligands targeting four distinct proteins.

The first target, phosphopantetheine adenylyltransferase (PPAT), is an essential homohexameric enzyme in coenzyme A biosynthesis that catalyzes the conversion of phosphopantetheine into dephospho‐coenzyme A [42]. The second, SARS‐CoV‐2 main protease (M^pro^), is a homodimeric cysteine protease whose catalytic residue Cys145 is targeted by covalent inhibitors [43]. We also studied Abelson tyrosine kinase 1 (Abl1), a kinase whose overactivation drives chronic myelogenous leukemia and is a well‐validated target for small‐molecule inhibitors [44]. Finally, we examined trypsin, a member of the serine proteinase family which plays an important role in digestion [45].

**FIGURE 4:**
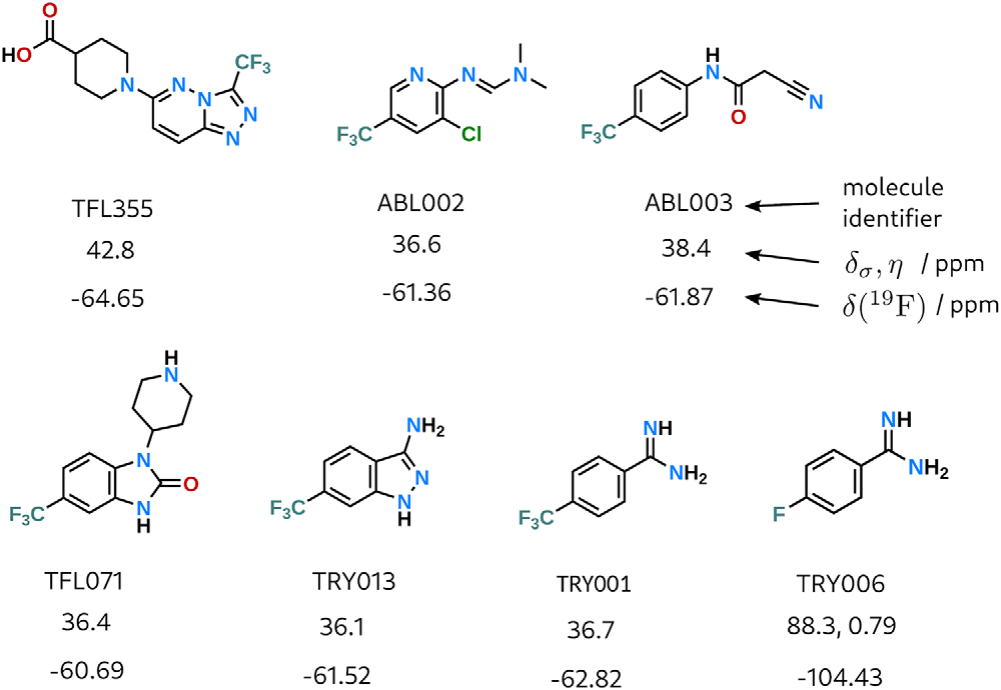
Molecular structures of ligands with dissociation constants ($K_{D}^{\text{CSA}}$) determined by the CSAKD method. The 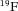 chemical shifts were measured in 50 mM phosphate buffer (pH 7.4) at 298.0 K, using indirect chemical shift referencing relative to 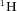. The CSA anisotropy and asymmetry ($\delta_{\sigma }$, η) were calculated via DFT.

The results from CSAKD affinity determination are shown in Table 1 along with $K_{D}$ values obtained from NMR titration and isothermal titration calorimetry (ITC) experiments. Experimental 3D structures to calculate the diffusion tensor are available for all targets. The diffusion is close to isotropic and therefore β has a small influence on $R_{\text{2,CSA}}$. Note that most of the ligands contain a ${\mathrm{CF}}_{3}$ with η=0, except for one trypsin ligand, TRY006, which contains a CF with an asymmetric 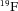 CSA tensor. The $K_{D}^{\text{CSA}}$ values show very good agreement, within standard deviation, with $K_{D}$ values from NMR titration and ITC experiments. Because the uncertainty in $K_{D}^{\text{CSA}}$ is determined by the unknown values for the parameters $S^{2}$, β, and γ, it remains of similar magnitude across different $K_{D}$ values. The relative error is therefore larger for low $K_{D}$ values, as illustrated here for TRY006. The chemical structures of the molecules from Table 1 are shown in Figure 4.

**TABLE 1:** $K_{D}^{\text{CSA}}$ values were fitted to the experimentally determined $\Delta R_{\text{2,obs}}^{\text{SL}}$ rates and the calculated $\Delta R_{\text{2,calc}}$ relaxation rates. In order to consider all possible values of the unknown parameters $S^{2}$, β and γ, an array of 8000 uniformly distributed values was generated with $S^{2}\in \left[0.5,0.85\right]$, β∈[0,π], and γ∈[0,π/2]. The $K_{D}^{\text{CSA}}$ values were determined by repeated fitting with these parameter combinations. The mean and standard deviation of the resulting $K_{D}^{\text{CSA}}$ values are reported. The theoretical $\Delta R_{\text{2,calc}}$ relaxation rates are calculated based on a model for anisotropic overall tumbling of the receptor. The effects of fast, that is, $\tau_{i}\ll \tau_{c}$, internal dynamics of the CSA tensor of the ligand in the bound state are treated by using the model free approach [20, 21]. An internal correlation time, $\tau_{i}$, of 1 ps was assumed for all ligands. The reported $\tau_{i}$ for ${\mathrm{CF}}_{3}$ groups in proteins is 17.2 ± 0.3 ps [40]. We note that $\tau_{i}$ values between 1 and 200 ps result in very similar $\Delta R_{\text{2,calc}}$ relaxation rates (see Supporting Information Figure S4). For isotropic tumbling, the Euler angles β and γ have no influence on the relaxation rates. Experimental relaxation data are from Rüdisser et al. [15]. The diffusion tensor components (D_x, y, z_) were calculated using the software HYDRONMR [19].

receptor	D_x, y, z_/ 10^−7^ s^−1^	ligand	$K_{D}$ ^#^/ μM	$\delta_{\sigma }$, η/ ppm	$\Delta R_{\text{2,f,obs}}^{\text{SL}}$/ s^−1^	$\Delta R_{\text{2,obs}}^{\text{SL}}$/ s^−1^	$K_{D}^{\text{CSA}}$/ μM
PPAT	0.262,0.253,0.256	TFL355	437.1 ± 66.5	42.8, 0	0.415^+^	1.49^+^	411 ± 92
M^pro^	0.285,0.358,0.393	TFL071	3187 ± 192	36.4, 0	0.360^+^	1.01^+^	3453 ± 553
		TRY013	6748 ± 237	36.1, 0	0.253^+^	0.761^+^	4433 ± 697
Abl1	0.407,0.465,0.582	ABL002	159	36.6, 0	0.601^$^	9.17^$^	114 ± 32
		ABL003	34	38.4, 0	1.4^$^	13.19^$^	72 ± 26
trypsin	1.25,1.37,1.54	TRY001	142 ± 6^*^	36.7, 0	0.252^+^	0.929^+^	361 ± 85
		TRY001	142 ± 6^*^	36.7, 0	0.554^$^	2.19^$^	365 ± 85
		TRY006	22 ± 2^##^	88.3, 0.79	0.250^+^	4.81^+^	38 ± 34

^#^ from 2D NMR titrations. ^*^ from DNP titrations [41] in Tris pH 8.0 instead of phosphate buffer pH 7.4 used here. ^##^ from ITC experiments, for attempts to measure TRY001 by ITC ‐ see Supporting Information. ^$^ data acquired at 11.75 T and 18.8 T. ^+^ data acquired at 11.75 T and 16.4 T.

## 3 Conclusion

Our newly developed CSAKD method enables accurate $K_{D}$ determination without isotopic labeling, using approximately 10 times less receptor and ligand than conventional NMR titration experiments [14]. Unlike other ligand‐observation methods (*e.g*., 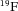 reporter assays [11], WaterLOGSY [12], or STD [13]), which require multiple ligand–receptor ratios, CSAKD operates with a single sample at a fixed concentration. While imaging STD NMR [46] also uses a single sample, it requires ligand concentrations ≥ 10 times higher than CSAKD due to sensitivity limitations. Although CSAKD is influenced by bound ligand dynamics and anisotropic receptor tumbling, we demonstrate experimentally and theoretically that these factors do not compromise accuracy. Moreover, CSAKD's sensitivity to ligand dynamics offers a novel approach to probe conformational entropy in the bound state. For ligands with known affinity, the order parameter ($S^{2}$) can be derived and correlated with entropy contributions [47], which modulates binding affinity. Existing models facilitate entropy estimation from $S^{2}$ [48, 49]. To improve accessibility, we developed a LEF‐based CSA predictor, eliminating reliance on computationally expensive DFT calculations. The LEF model achieves a RMSE of less than 2 ppm for CF and ${\mathrm{CF}}_{3}$ groups, corresponding to less than 5% error in the calculated $R_{2,\text{CSA}}$ relaxation rates, while offering a 7000‐fold acceleration in computational speed over DFT. This advancement makes CSAR [15] and CSAKD accessible to a wide range of laboratories.

In summary, we present CSAKD, a novel 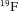 NMR‐based method for the rapid and titration‐free determination of $K_{D}$ values at low μM receptor concentrations. The integration of a new machine learning approach for predicting CSA tensors makes CSAKD widely accessible, as it eliminates the need for extensive computational infrastructure. CSAKD integrates seamlessly into the FBDD pipeline, enabling direct affinity‐based prioritization of 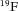 NMR screening hits.

## Conflicts of Interest

The authors declare no conflicts of interest.

## Supporting information


**Supporting File 1**: anie73059‐sup‐0001‐SuppMat.pdf.

**Supporting File 2**: anie73059‐sup‐0002‐Data.zip.

## Data Availability

The data that support the findings of this study are available from the corresponding author upon reasonable request.
