# Supplementary material for: CSAKD: Determining Absolute Ligand Affinities From 19F NMR Chemical Shift Anisotropy
Source: Angew Chem Int Ed Engl. 2026 Jun 12;65(33):e6036832. doi: 10.1002/anie.6036832 (PMC13452527; doi:10.1002/anie.6036832)
Supplement: Supplementary file 1 [file ANIE-65-e6036832-s001.pdf]

Supplementary information

CSAKD: Determining Absolute Ligand Affinities from ^{19}F NMR Chemical Shift Anisotropy

Simon H. Rüdisser · Gabriela Stadler · Alvar D. Gossert

Simon H. Rüdisser
E-mail: simon.ruedisser@biol.ethz.ch
Alvar D. Gossert
E-mail: alvar.gossert@biol.ethz.ch
ETH Zürich, 8093 Zürich, Switzerland

Table S1 Symbols and definitions

γ_{F}	gyromagnetic ratio of ^{19}F
γ_{H}	gyromagnetic ratio of ^{1}H
$\hbar$	reduced Planck constant $(h/(2\pi))$
μ_0	magnetic permeability in the vacuum
β	Euler angle for the rotation around D_x
γ	Euler angle for the rotation around $\overrightarrow{\sigma_{zz}}$
CSA	chemical shift (or shielding) anisotropy
σ	chemical shielding tensor
σ_{xx}	magnitude of the x component of σ in the principal component frame
σ_{yy}	magnitude of the y component of σ in the principal component frame
σ_{zz}	magnitude of the z component of σ in the principal component frame
$\overrightarrow{\sigma_{xx}}$	eigenvector for the σ_{xx} component
$\overrightarrow{\sigma_{yy}}$	eigenvector for the σ_{yy} component
$\overrightarrow{\sigma_{zz}}$	eigenvector for the σ_{zz} component
$\Delta\sigma$	chemical shielding anisotropy
η	chemical shielding asymmetry
σ_{iso}	isotropic chemical shielding
δ_σ	reduced anisotropy of the chemical shift tensor
$\delta_\sigma^{\mathrm{eff}}$	effective δ_σ
R_2	Transverse relaxation rate
$R_{2,DD}$	Transverse dipole-dipole relaxation rate
$R_{2,\mathrm{CSA}}$	Transverse CSA relaxation rate
$R_{2,\mathrm{eff}}$	Transverse relaxation rate with contributions from CSA, DD and chemical exchange
R_{ex}	Transverse relaxation due to chemical exchange
$R_{2,\mathrm{DD,b}}$	Transverse dipole-dipole relaxation rate of the ligand in the bound state
$R_{2,\mathrm{CSA,b}}$	Transverse CSA relaxation rate of the ligand in the bound state
R_2^{SL}	Transverse relaxation rate under spin-lock conditions
ΔR_2^{SL}	Difference of transverse relaxation rate under spin-lock conditions at two different B_0 fields
$R_{2,\mathrm{CSA,b},B_{0_1}}$	Transverse CSA relaxation rate in the bound state at field B_{0_1}
B_{0_1}	external magnetic field 1
B_{0_2}	external magnetic field 2
$[R]$	receptor concentration
$[R]_0$	total receptor concentration
$[L]$	ligand concentration
$[L]_0$	total ligand concentration
p_{b}	fraction bound ligand
p_{f}	fraction free ligand
K_{D}	dissociation constant
D_x, D_y, D_z	diffusion tensor components in the principal axis frame
ζ	diffusion tensor anisotropy
η_{D}	diffusion tensor asymmetry
τ_{c}	rotational correlation time
τ_{i}	internal correlation time
S^2	model free order parameter

1 List of associated files

Table S2 Description of files and computer programs which are provided in the supplementary information or at https://github.com/SimonRuedisser/19F_NMRscreening.

filename	software	description
LEF.ipynb	Jupyter notebook	LEF-based CSA predictor
CSAKD.ipynb	Jupyter notebook	$R_{2,\mathrm{CSA}}$ simulation and $K_\mathrm{D}^\mathrm{CSA}$ determination
CSA_CF3.xlsx	MS Excel	CSA values for CF_3 molecules calculated by DFT methods
CSA_CF.xlsx	MS Excel	CSA values for CF molecules calculated by DFT methods
OpenFL600_eff_delta_sigma_CF3.xlsx	MS Excel	$\delta_\sigma^\mathrm{eff}$ values for CF_3 molecules from RF$^\#$ prediction
OpenFL600_eff_delta_sigma_CF.xlsx	MS Excel	$\delta_\sigma^\mathrm{eff}$ values for CF molecules from RF$^\#$ prediction

$^\#$ . . . random forest regression

2 Theory

2.1 Chemical Shift (Shielding) Anisotropy (CSA)

In an anisotropic CSA tensor, the magnitude of the shielding in the z direction, σ_{zz}, is different than the magnitude of the shielding in the x- or y- directions (σ_{xx}, σ_{yy}). If the magnitudes of shielding in the x- and y-directions are different, the shielding is asymmetric. The anisotropy $\Delta\sigma$ and asymmetry η of the shielding tensor are defined by equations S1 according to the "Haeberlen–Mehring–Spiess" convention[1]. Especially in solid-state NMR, an alternative notation is commonly used to describe the anisotropy and asymmetry as defined in equation S2.

$$\begin{aligned} &\Delta\sigma = \sigma_{zz} - \frac{\sigma_{xx} + \sigma_{yy}}{2} \\ &\mid \sigma_{zz} - \sigma_{iso} \mid \geq \mid \sigma_{yy} - \sigma_{iso} \mid \geq \mid \sigma_{xx} - \sigma_{iso} \mid \\ &\eta = \frac{\sigma_{yy} - \sigma_{xx}}{\sigma_{zz} - \sigma_{iso}} \\ &\sigma_{iso} = \frac{\sigma_{xx} + \sigma_{yy} + \sigma_{zz}}{3} \end{aligned} \tag{S1}$$

$$\begin{aligned} &\delta_\sigma = \sigma_{zz} - \sigma_{iso} \\ &\delta_\sigma = \frac{2}{3}\Delta\sigma \end{aligned} \tag{S2}$$

2.2 The effective anisotropy, a combined descriptor of δ_σ and η

The relaxation rate $R_{2,\mathrm{CSA}}$ depends on the anisotropy, δ_σ, and asymmetry, η as shown in Equation S3. For building a machine learning model to predict the shape of the CSA tensor, a single parameter, which combines anisotropy and asymmetry is advantageous. We therefore introduce the effective $\Delta\sigma$, $\delta_\sigma^{\mathrm{eff}}$ which is defined by Equation S4. $\delta_\sigma^{\mathrm{eff}}$ can be directly used to calculate $R_{2,\mathrm{CSA}}$ according to Equation S3.

$$R_{2,\mathrm{CSA}} = \frac{1}{20}\gamma_{\mathrm{F}}^2 B_0^2 \delta_\sigma^2 \left(1 + \frac{\eta^2}{3}\right)\left(4\tau_c + \frac{3\tau_c}{1 + \omega_{\mathrm{F}}^2\tau_c^2}\right) \tag{S3}$$

$$\delta_\sigma^{\mathrm{eff}} = \delta_\sigma \sqrt{\left(1 + \frac{\eta^2}{3}\right)} \tag{S4}$$

For CF_3 groups, averaging of the three CSA tensors results in an oblate spheroid with the short axis along the C_3 rotation axis[2]. The two tensor components which are perpendicular to the C_3 axis have equal magnitude. It can be show that for an oblate spheroid tensor, $\Delta\sigma$ calculated with the zz-component along C_3, is equivalent to the expression given in Equation S4 for a tensor with the zz-component along one of the long axes (see Equation S5).

$$\delta_\sigma^{\mathrm{eff}} = \delta_\sigma^{C_3 \| \sigma_{zz}} = \delta_\sigma \sqrt{\left(1 + \frac{\eta^2}{3}\right)} \tag{S5}$$

2.3 CSA relaxation for the case of anisotropic tumbling

The $R_{2,\mathrm{CSA}}$ relaxation rate for a molecule which is undergoing axial symmetric diffusion is given by Equation S6 to S13[3].

$$R_{2,\mathrm{CSA}} = \frac{1}{40}\gamma_{\mathrm{F}}^2 B_0^2 \delta_\sigma^2 \sum_{i=0}^{2} c_i \left[3\tilde{J}_i(\omega_0) + 4\tilde{J}_i(0)\right] \tag{S6}$$

$$\tilde{J}_i(\omega) = \frac{2\tau_i}{1+\omega^2\tau_i^2} \tag{S7}$$

$$\frac{1}{\tau_0} = 6D_\perp \tag{S8}$$

$$\frac{1}{\tau_1} = D_\parallel + 5D_\perp \tag{S9}$$

$$\frac{1}{\tau_2} = 4D_\parallel + 2D_\perp \tag{S10}$$

$$c_0 = \frac{1}{4}\left[(3\cos^2\beta - 1) + \eta\ \sin^2\beta\ 2\ \cos 2\gamma\right]^2 \tag{S11}$$

$$c_1 = \frac{1}{3}\sin^2\beta\left[\cos^2\beta(3-\eta\cos 2\gamma)^2 +\ \eta^2\sin^2 2\gamma\right] \tag{S12}$$

$$c_2 = \left[\sqrt{\frac{3}{4}}\sin^2\beta + \left[\frac{\eta}{2\sqrt{3}}\right](1+\cos^2\beta)\cos 2\gamma\right]^2 + \frac{\eta^2}{3}\sin^2 2\gamma\cos^2\beta \tag{S13}$$

If σ_{zz} is parallel to D_z ($\beta = 0$), $c_0 = 1$, $c_1 = 0$ and $c_2 = \eta^2/3$. For $\eta = 0$ the rate becomes independent of γ and reduces to the isotropic solution. Note that $\omega_0 = \gamma_{\mathrm{F}} B_0$.

2.4 Dipole-dipole relaxation for the case of anisotropic tumbling

The dipolar relaxation of spin-1/2 nuclei depends on the distance (r) between the two nuclei involved. The dipole-dipole relaxation rate can be calculated from the spectral densities, $J(\omega)$, using Equation S14[4, 5]. γ_{F} and γ_{H} are the gyromagnetic ratios of the nuclei, which in this example are ^{19}F and ^{1}H.

$$R_{2,\mathrm{DD}} = \frac{1}{20}\hbar^2\mu_0^2\gamma_{\mathrm{F}}^2\gamma_{\mathrm{H}}^2 \sum_i \frac{1}{r^6_{(\mathrm{F},\mathrm{H}_i)}}\left[4J(0) + 3J(\omega_{\mathrm{F}}) + 6J(\omega_{\mathrm{H}}) + J(\omega_{\mathrm{F}}-\omega_{\mathrm{H}}) + 6J(\omega_{\mathrm{F}}+\omega_{\mathrm{H}})\right] \tag{S14}$$

For an axially symmetric molecule with only two rotational diffusion coefficients parallel $D_\parallel$ and perpendicular $D_\perp$ to the unique axis of the molecule, the spectral densities, $J(\omega)$, are given by Equation S15. θ is the angle between the F—H internuclear vector and unique axis of the molecule. The correlation times τ_1, τ_2 and τ_3 depend on the rotational diffusion coefficients (Equations S16 to S18).

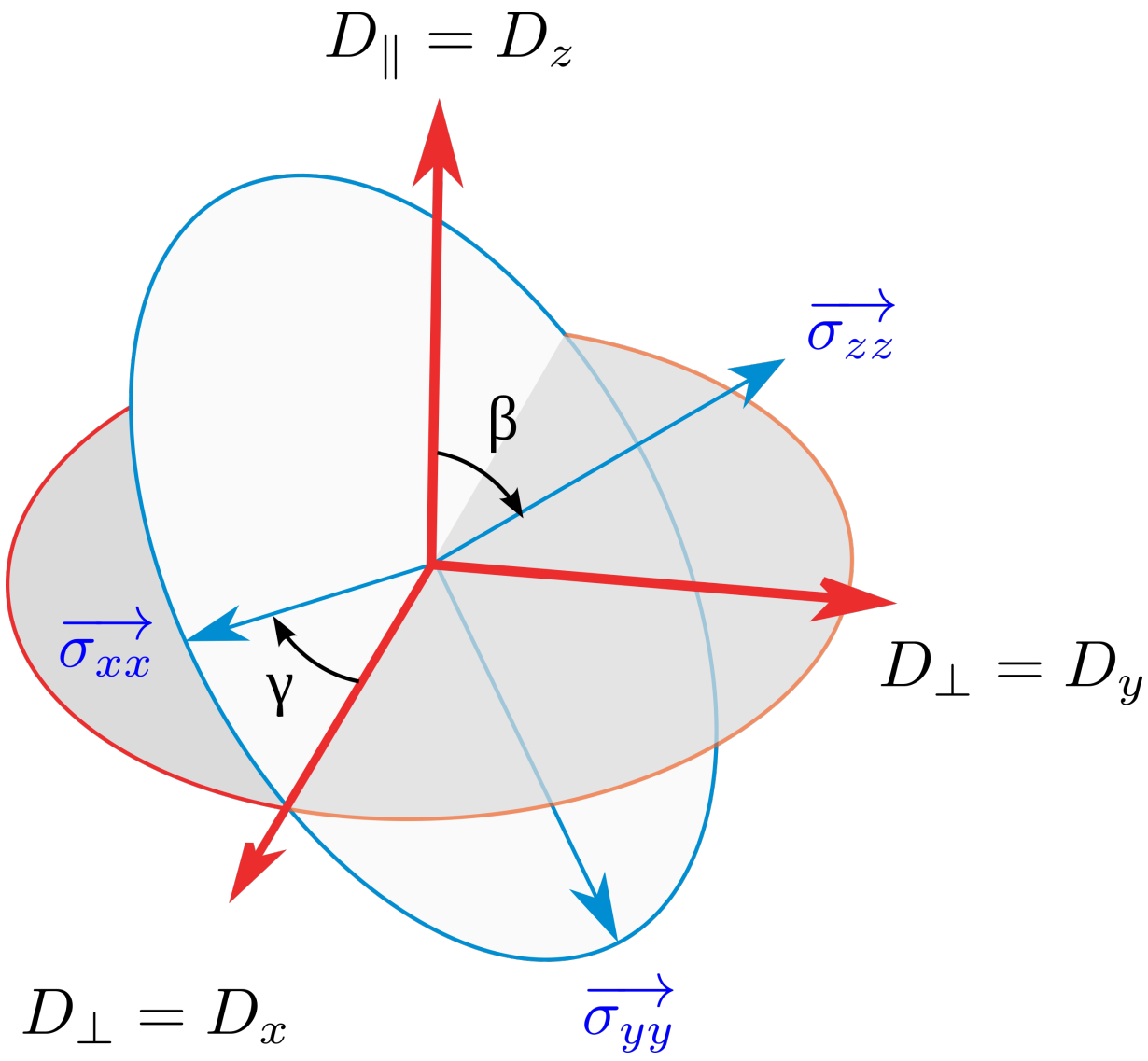

Fig. S1 Definitions of the Euler angles β and γ. Since the diffusion tensor is a symmetric top, *i.e.* $D_x = D_y$, only the two Euler angles β and γ are required to define the rotation (Figure adapted from `https://en.wikipedia.org/wiki/Euler_angles`).

$$J_i(\omega) = \sum_{i=1}^{3} C_i \left[\frac{2\tau_i}{1+\omega^2\tau_i^2}\right] \tag{S15}$$

$$\frac{1}{\tau_1} = 4D_\| + 2D_\perp \tag{S16}$$

$$\frac{1}{\tau_2} = D_\| + 5D_\perp \tag{S17}$$

$$\frac{1}{\tau_3} = 6D_\perp \tag{S18}$$

$$C_1 = \frac{3}{4}\sin^4\theta \tag{S19}$$

$$C_2 = 3\sin^2\theta\cos^2\theta \tag{S20}$$

$$C_3 = \frac{1}{4}(3\cos^2\theta - 1)^2 \tag{S21}$$

Equation S22 describes the case for isotropic tumbling, where a single rotational correlation time, τ_c, suffices to describe the rotational diffusion.

$$R_{2,\mathrm{DD}} = \frac{1}{10}\hbar^2\mu_0^2\gamma_\mathrm{F}^2\gamma_\mathrm{H}^2 \sum_i \frac{1}{r^6_{(\mathrm{F},\mathrm{H}_i)}} \left[\tau_c + \frac{3\tau_c}{1+\omega_\mathrm{F}^2{\tau_c}^2} + \frac{\tau_c}{1+(\omega_\mathrm{F}-\omega_\mathrm{H})^2\tau_c^2} + \frac{6\tau_c}{1+(\omega_\mathrm{F}+\omega_\mathrm{H})^2\tau_c^2}\right] \tag{S22}$$

2.5 Internal motion

Internal motion can be described by the model free approach. The resulting spectral densities are given by Equation S23. S^2 is the generalized order parameter which describes the magnitude of internal motion on a cone and τ_i is the internal correlation time[6, 7]. Note that $\tau_i \ll \tau_c$.

$$J_n(\omega) = 2\left[\frac{S^2\tau_n}{1+(\omega\tau_n)^2} + \frac{(1-S^2)\tau_n^{'}}{1+(\omega\tau_n^{'})^2}\right] \tag{S23}$$

The correlation times are given by Equations S24.

$$\begin{aligned} \tau_0^{'} &= \frac{\tau_0\tau_i}{\tau_0+\tau_i} \\ \tau_1^{'} &= \frac{\tau_1\tau_i}{\tau_1+\tau_i} \\ \tau_2^{'} &= \frac{\tau_2\tau_i}{\tau_2+\tau_i} \end{aligned} \tag{S24}$$

3 Simulation of $\Delta R_{2,\mathrm{CSA}}$

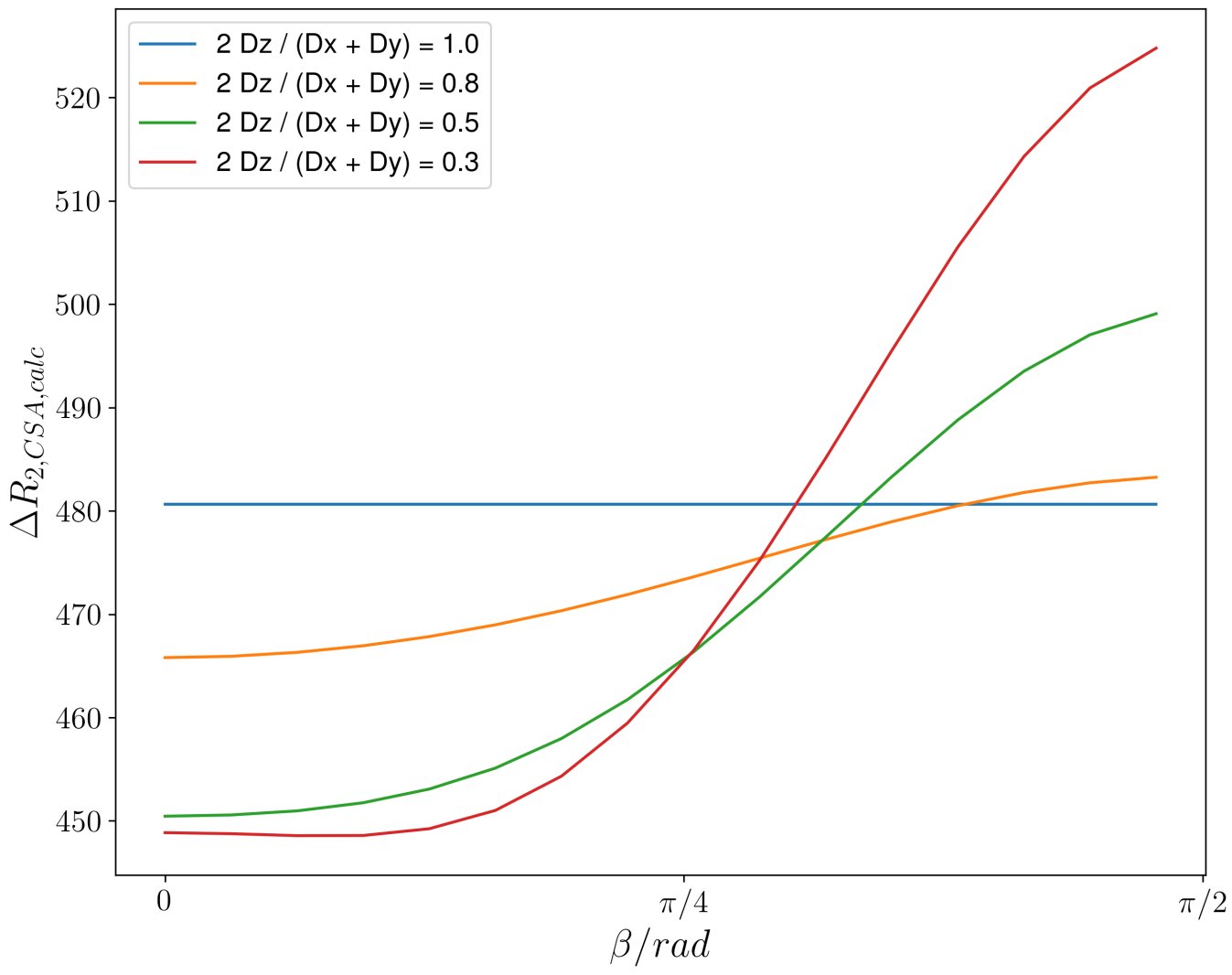

Fig. S2 $\Delta R_{2,\mathrm{CSA}}$ is shown as a function of the Euler angle β for various diffusion tensor anisotropies $\eta_D = 2D_z/(D_x+D_y)$. The following parameters have been used for the calculations: $\delta_\sigma = 69.5$ ppm, $\eta = 0.998$ ppm, $D_x + D_y + D_z = 3 \times 0.25$ 10^7 s^{-1}, $\tau_i = 1$ ps, $\gamma = 54°$, $B_{0_1} = 16.4$ T, $B_{0_2} = 11.75$ T.

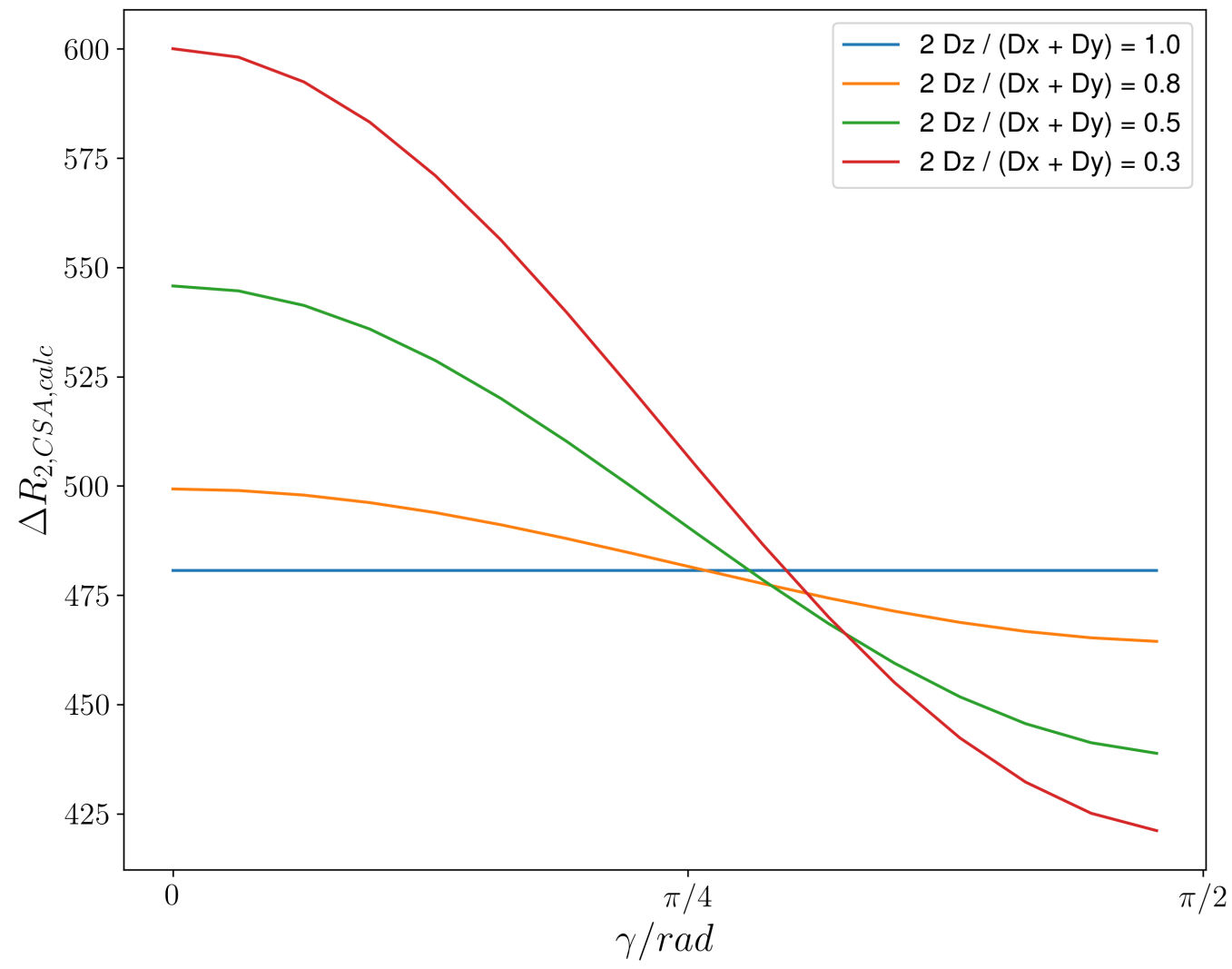

Fig. S3 $\Delta R_{2,\mathrm{CSA}}$ is shown as a function of the Euler angle γ for various diffusion tensor anisotropies $\eta_D = 2D_z/(D_x+D_y)$. The following parameters have been used for the calculations: δ_σ = 69.5 ppm, η = 0.998 ppm, $D_x + D_y + D_z = 3 \times 0.25$ 10^7 s^{-1}, τ_i = 1 ps, $\beta = 54°$, B_{0_1} = 16.4 T, B_{0_2} = 11.75 T.

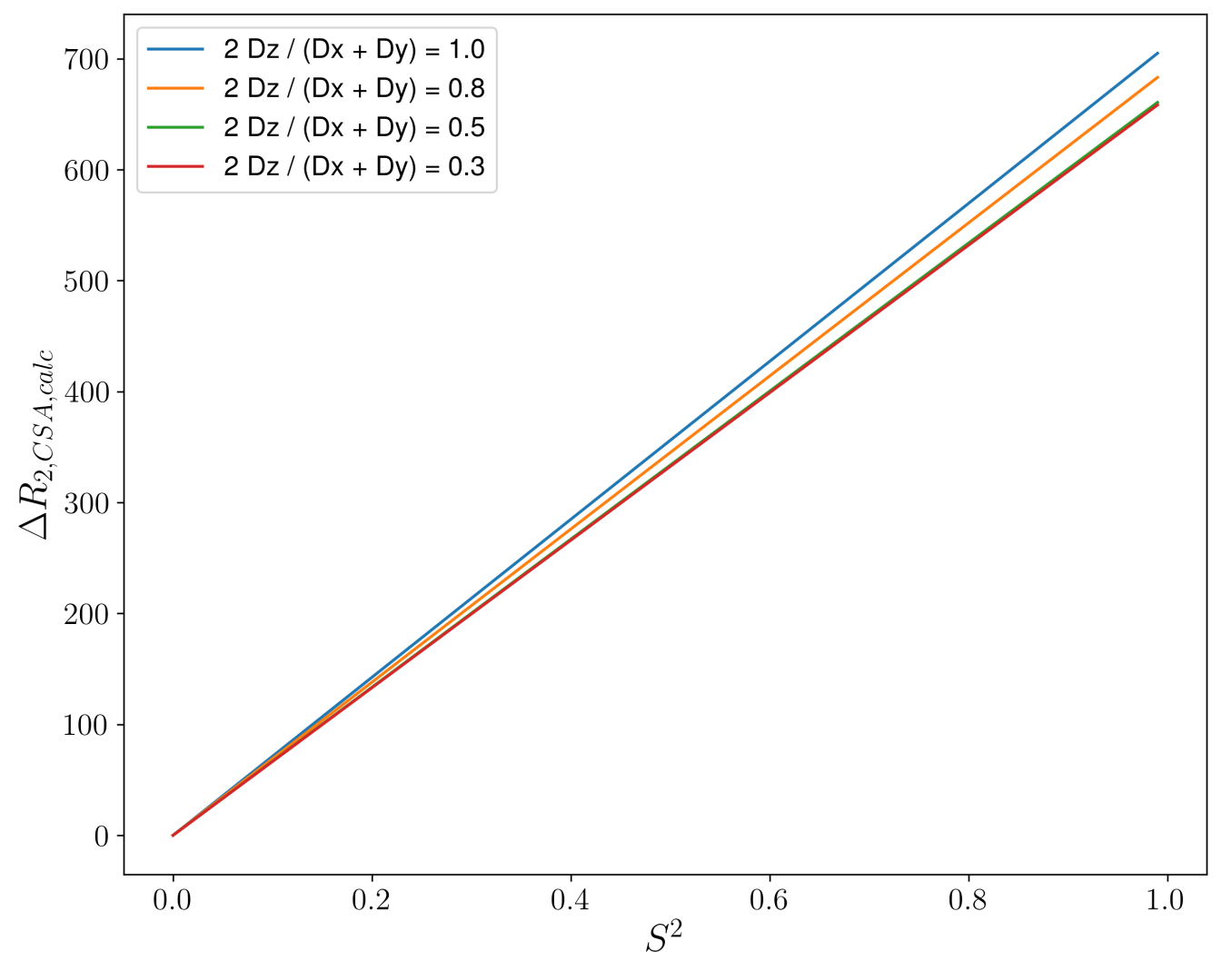

Fig. S4 $\Delta R_{2,\mathrm{CSA}}$ is shown as a function of the order parameter S^2 for various diffusion tensor anisotropies $\eta_D = 2D_z/(D_x+D_y)$. The following parameters have been used for the calculations: δ_σ = 69.5 ppm, η = 0.998 ppm, $D_x+D_y+D_z$ = 3×0.25 10^7 s^{-1}, τ_i = 1 ps, $\beta = \gamma = 0$, B_{0_1} = 16.4 T, B_{0_2} = 11.75 T.

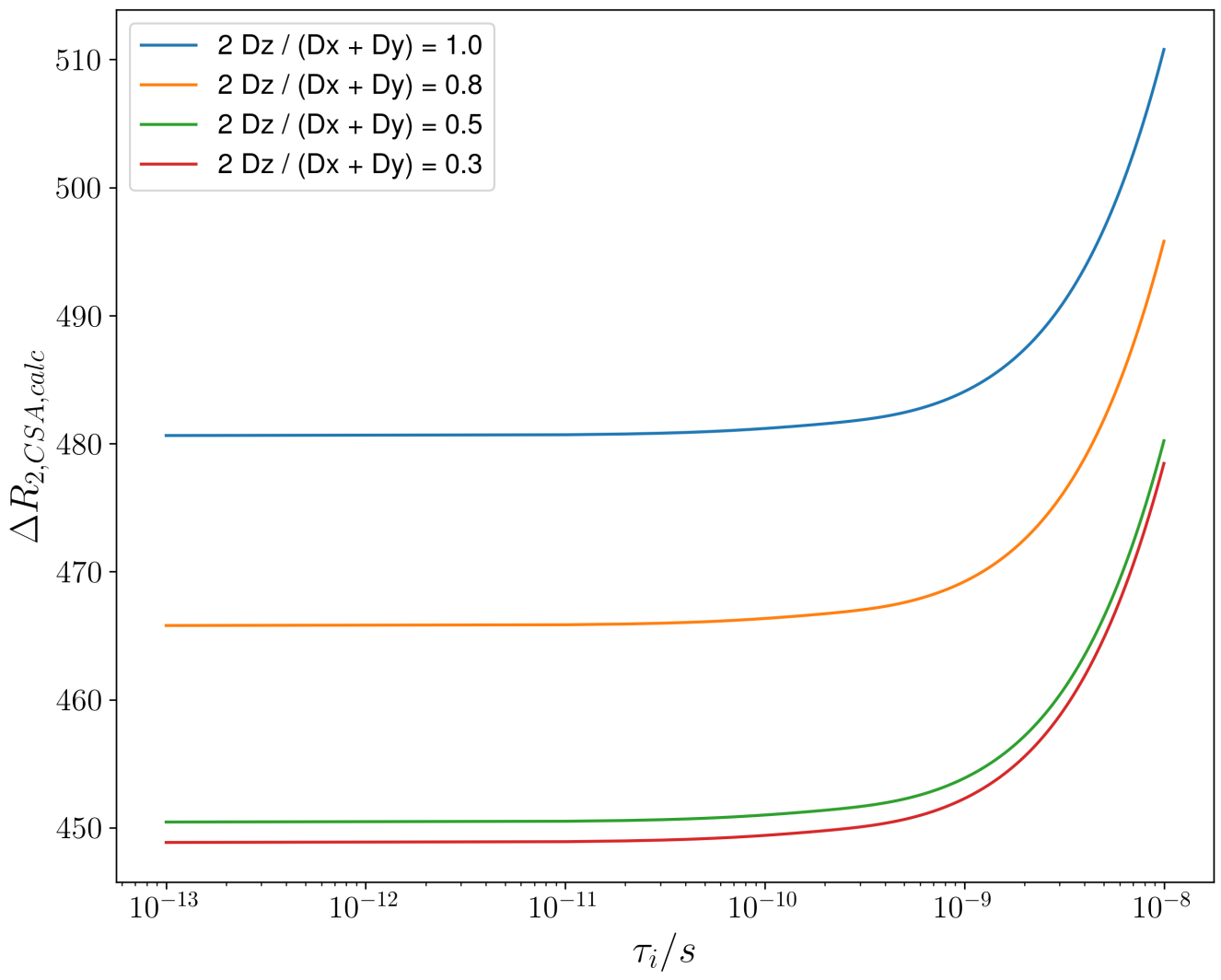

Fig. S5 $\Delta R_{2,\mathrm{CSA}}$ is shown as a function of the internal correlation time τ_i for various diffusion tensor anisotropies $\eta_D = 2D_z/(D_x + D_y)$. The following parameters have been used for the calculations: $\delta_\sigma = 69.5$ ppm, $\eta = 0.998$ ppm, $D_x + D_y + D_z = 3 \times 0.25\ 10^7$ s^{-1}, $S^2 = 0.675$, $\beta = \gamma = 0$, $B_{0_1} = 16.4$ T, $B_{0_2} = 11.75$ T.

4 Diffusion tensor calculations with HYDRONMR

HYDRONMR[8] for Linux was downloaded from https://leonardo.inf.um.es/macromol/programs/hydronmr/hydronmr.htm.
Coordinates from www.rcsb.org were prepared for HYDRONMR calculations by removing water molecules and ligands. Crystallography related copies of the monomer were created for the PPAT hexamer. The following pdb depositions were used: trypsin: 1bty, PPAT: 6chq, Mpro: 7cam, Abl1: 2src. The parameters for HYDRONMR are given in Table S3.

Table S3 Parameters for HYDRONMR computations.

3.1	AER, radius of the atomic elements
6	NSIG
2.0	Minimum radius of beads in the shell (SIGMIN)
3.0	Maximum radius of beads in the shell (SIGMAX)
298	T (temperature, K)
0.01	ETA (Viscosity of the solvent in poises)
1	Iflag (dipoles)

5 Protein expression and purification

5.1 PPAT (CoaD)

PPAT was expressed in *E. Coli*. See Rüdisser *et al.* 2026 for a detailed description of protein expression and purification.

5.2 M^{pro}

M^{pro} was expressed in *E. Coli* fully protonated with a uniform ^{15}N and $^{13}C^{\epsilon}$ methionine labeling pattern. See Rüdisser *et al.* 2026 for a detailed description of protein expression and purification.

5.3 Expression and purification of unlabeled Abelson tyrosine kinase (Abl1)

Abelson tyrosine kinase (Abl1) was expressed with an N-terminal His_6-tag (green) followed by a human rhinovirus (HRV) 3C protease cleavage site (red) as shown in the sequence below. The sequence of the purified protein after cleavage by HRV-3C protease (bold fraction) corresponds to the sequence of the catalytic domain cassette (SH3-SH2-SH1) 42–44 of human Abl1 (blue, UniProt ID: P00519[64-515]) with two additional residues added to the N-terminus. Abl1 was co-expressed together with untagged protein tyrosine phosphatase 1b (PTP1b, UniProt ID: P18031[1-283]) on a pCDFDuet-1 plasmid. Co-expression of PTP1b served to counteract activation of Abl1 by autophosphorylation 42,52 , thereby highly increasing yield and homogeneity of the protein preparation. The plasmid used for expression of Abl1 encoded for the same sequence as used by Skora *et al.*[9, 10] for their CF_3 fragment library screening and was obtained as a gift from Novartis Pharma AG.

MGSSHHHHHHLEVLFQGPNLFVALYDFVASGDNTLSITKGEKLRVLGYNHNGEWCEAQTKNGQG
WVPSNYITPVNSLEKHSWYHGPVSRNAAEYLLSSGINGSFLVRESESSPGQRSISLRYEGRVYHYRI
NTASDGKLYVSSESRFNTLAELVHHHSTVADGLITTLHYPAPKRNKPTVYGVSPNYDKWEMERTD
ITMKHKLGGGQYGEVYEGVWKKYSLTVAVKTLKEDTMEVEEFLKEAAVMKEIKHPNLVQLLGV
CTREPPFYIITEFMTYGNLLDYLRECNRQEVNAVVLLYMATQISSAMEYLEKKNFIHRDLAARNCL
VGENHLVKVADFGLSRLMTGDTYTAHAGAKFPIKWTAPESLAYNKFSIKSDVWAFGVLLWEIAT
YGMSPYPGIDLSQVYELLEKDYRMERPEGCPEKVYELMRACWQWNPSDRPSFAEIHQAFETMFQ
ESSISDEVEKELGKQGV

E. coli BL21 Star (DE3) competent cells (Thermo Fisher Scientific) were transformed with plasmid DNA coding for Abl1 and PTP1b and were incubated overnight at 37°C on an agar plate containing streptomycin (stp, 100 mg/l). For the preculture, 50 ml of Luria Bertani (LB) medium containing stp was inoculated with four colonies and incubated at 37°C for 20 h in a 500 ml baffled Erlenmeyer flask at 100 rpm shaking speed in an Infors HT Multitron shaking incubator. The optical density at 600 nm (OD 600) of the preculture was measured and 28 ml of the preculture was transferred to 1 l of LB medium containing stp in order to reach a start OD 600 of 0.1. The main culture was grown at 37°C in a 5 l baffled Erlenmeyer flask at 95 rpm shaking until OD 600 reached 0.7. The shaker was cooled to 18°C, and after an equilibration time of 30 min the culture was induced with 1 mM isopropyl β-d-thiogalactopyranoside (IPTG). The culture was grown at 18°C for 18 h at 95 rpm. Cells were harvested by centrifugation at 4'000 g for 10 min at 4°C (Beckmann Coulter Avanti JXN-26 Centrifuge equipped with JLA8.1000 rotor) and stored at -80°C.

The cell pellet was thawed on ice and resuspended in 25 ml lysis buffer (50 mM Tris pH 8.0, 300 mM NaCl, 5 mM imidazole, 10% glycerol, 1 mM DTT, one spatula tip of DNAse I, 1 mM PMSF). Cells were lysed with M-110L Microfluidizer (Microfluidics) in 4 cycles at 15'000 PSI. The obtained lysate was centrifuged at

35'000 g for 45 min at 4°C (Beckmann Coulter Avanti JXN-26 Centrifuge equipped with JLA25.50 rotor). The resulting supernatant was filtered through a 210.45 µM syringe filter and loaded onto a 5 ml Ni-NTA Superflow Cartridge (Qiagen) using ÄKTAxpress (GE Healthcare). The column was washed with binding buffer (50 mM Tris pH 8.0, 300 mM NaCl, 5 mM imidazole, 10% glycerol, 1 mM DTT) and eluted in a one step elution with elution buffer (50 mM Tris pH 8.0, 300 mM NaCl, 250 mM imidazole, 10% glycerol, 1 mM DTT). Buffer was exchanged back into binding buffer using a HiPrep 26/10 Desalting column. Purity and concentration of the protein were monitored by RP-HPLC using a continuous gradient from 80% buffer A (0.1% TFA inH_2O) and 20% buffer B (0.1% TFA in 90% MeCN, 10% H_2O) to 100% buffer B (Agilent 1100 Series equipped with POROS C8 column at 80°C and UV 215 detector). Cleavage of His_6-tag was performed overnight using 0.7 mg of HRV-3C protease (His_6-tagged, lab-internal production). Abl1 was separated from HRV-3C protease and cleaved tag by a second round of Nickel Immobilized Metal Affinity Chromatography (Ni-IMAC), where the target protein was collected in the flow-through. The protein was concentrated using a 30 kDa concentrator (Amicon Ultra-15, Merck Millipore) and was further purified by size exclusion chromatography (SEC) in SEC buffer (50 mM Tris pH 8.0, 200 mM NaCl, 5% glycerol, 2 mM DTT) through a Superdex 75 16/600 column. The purified protein was concentrated to 30 µM using a new 30 kDa concentrator. Purity, concentration and yield were determined by RP-HPLC and NanoDrop ND-1000 Spectrophotometer (Thermo Scientific). The protein was aliquoted, flash frozen in liquid nitrogen and stored at -80°C.

5.4 trypsin

Samples had been prepared with trypsin from bovine pancreas (Merck).

6 NMR spectroscopy

6.1 NMR instrumentation

A probe with separate ^{19}F and ^{1}H channels (19F/1H) allows ^{1}H decoupling during ^{19}F acquisition, yielding a single line of high sensitivity. Therefore, measurements of the CF containing ligands were carried out on QCI-F Cryoprobes. The NMR experiments were performed on the following Bruker instruments: 600 MHz AVIIIHD equipped with a QCI 1H/19F-13C/15N-2H Cryoprobe (for 19F/1H double resonance experiments, *i.e.* decoupling), 500 MHz AVNEO equipped with a QCI 1H&19F-13C/15N/31P-2H Cryoprobe (tunable for ^{19}F or ^{1}H single resonance experiments) and 700 MHz AVNEO equipped with a QCI 1H/19F-13C/15N-2H Cryoprobe. Titrations and screening experiments were measured on the 600 MHz AVIIIHD spectrometer. All measurements were performed in NMR tubes with 3 mm diameter, at 298 K.

6.2 ^{19}F NMR relaxation experiments

Measurements of transverse relaxation rates were performed using simple $T_1\rho$-type experiments recorded as a pseudo2D in interleaved acquisition to avoid differential heating between scans with relaxation delays between 2.5 and 250 ms. Spin lock strengths between 8 and 10 kHz were applied in these experiments (ω_{SL} = 8 or 10 kHz). The pulse sequence is available in the supplementary material of Rüdisser *et al.*[2]. The experimental conditions for $T_1\rho$-type experiments for specific receptor molecules are provided in Table S4.

Table S4 Experimental conditions for relaxation rate measurements for CSAR.

receptor	c(receptor) / μM	c(ligand) / μM	T / K	buffer
M^{pro}	30	200	298.0	45 mM sodium phosphate pH 7.55, 45 mM NaCl, 1 mM DTT
PPAT	5	200	298.0	18 mM sodium phosphate pH 7.0, 1 mM DTT
trypsin	20^{+}	200	298.0	PBS pH 7.4
Abl1	20	200	298.0	45 mM Tris pH 8.0, 180 mM NaCl, 4.5% glycerol

All buffers contain 10 % D_2O (Merck 99.8% 617385-1.107KG), 50 µM TFA (trifluor acetic acid, Acros Organics 99.5% 293811000 Lot: A0417100), 100 µM DSS-D_6 (sodium trimethylsilylpropanesulfonate, CIL 98 % DLM-8206-1).
DTT: dithiothreitol, Merck $\geq$ 98 % D0632-10G,
PBS: Gibco 10010-015,
glycerol-deuterated 99+%: Acros Organics, 158920010
$^{+}$ for TRY006, the concentration of trypsin has been recalculated after auto-proteolysis for 7 hours according to the rate provided in [2] to 8 µM.

6.3 K_{D} determination by 2D NMR titration experiments for PPAT ligands

XL-ALSOFAST-[^{13}C,^{1}H]-HMQC[11] spectra of 50 µM $^{13}C^{\epsilon}$ labeled PPAT in 18 mM sodium phosphate pH 7.0, 1 mM DTT, 10% D_2O in the presence of up to 1 mM ligand were recorded. Spectra were recorded at 308 K with 64 scans in 1.5 h on a 600 MHz Avance IIIHD spectrometer. The chemical shift was fitted to a single-binding-site model.

6.4 K_D determination by 2D NMR titration experiments for M^{pro} ligands

XL-ALSOFAST-[^{13}C,^{1}H]-HMQC[11] spectra of 75 µM $^{13}C^{\epsilon}$ labeled M^{pro} in 45 mM sodium phosphate pH 7.0, 45 mM sodium phosphate, 1 mM DTT, 10% D_2O in the presence of up to 1 mM ligand were recorded. Spectra were recorded at 298 K with 32 scans in 1.5 h on a 700 MHz Avance NEO spectometer. The chemical shift was fitted to a single-binding-site model.

6.5 K_D determination by 2D NMR titration experiments for Abl1 ligands

The K_D values for ABL002 and ABL003 are 159 µM and 34 µM, respectively. In biochemical assays, the half-maximal inhibitory concentrations (IC_{50}) were >800 µM and 45 µM, respectively. These values are unpublished data from Novartis Pharma AG.

6.6 NMR data processing

Data were processed using TopSpin 4.0.7. Peak picking of spectra was done with the nmrglue module for Python3 (Helmus and Jaroniec 2013, https://www.nmrglue.com). Fitting of relaxation data was performed with self-written Python3 scripts (www.python.org) using the scipy (www.scipy.org), numpy (www.numpy.org) and pandas (www.pandas.pydata.org) modules. The experimental conditions for specific receptor molecules are provided in Table S4.

7 ^{19}F CSA calculations for screening library molecules

7.1 ^{19}F CSA calculation with DFT methods

The three-dimensional structures of the small molecules have been generated from SMILES using openbable (www.openbable.org). Energy minimization was performed using the minimax minimizer (Witnotp)[12] and the Tripos all atom force field (TAFF)[13].

DFT calculations were performed using Gaussian 09[14], following a protocol similar to that described by Rüdisser *et al.*[15]. To enhance computational efficiency, several modifications to the original protocol were made. Geometry optimizations were conducted at the M06L/def2TZVPP level of theory[16, 17], utilizing density fitting and an SMD implicit solvation model[18] to account for the dielectric effects of the surrounding water.

Chemical shielding tensors were computed at the energy minima using the Gauge-Independent Atomic Orbital (GIAO) method [19, 20], employing the M06 hybrid functional. The cc-pVTZ basis set was used for carbon (C), hydrogen (H), nitrogen (N), and oxygen (O), while the AUG-cc-pVTZ basis set was used for fluorine (F). For CF_3 groups, individual tensors for the fluorine atoms were averaged by static tensor averaging, as described in the supplementary information by Rüdisser *et al.*[15]. To compute the eigenvalues for the CSA, σ_{ii}, where $ii \in \{xx, yy, zz\}$ for CF_3 group, the components of the three tensors, σ_{ij} with $i, j \in \{x, y, z\}$, were averaged, and the eigenvalues of the resulting tensor were calculated. These computations were carried out using a self-written Python 3 script (www.python.org) utilizing the numpy module (www.numpy.org).

The CSA tensors have been computed for 187 CF_3 and for 324 CF fragments from the screening library using the EULER cluster computing infrastructure at the ETH Zürich (https://scicomp.ethz.ch/wiki/Euler). These results are used as training and test sets for random forest regression based on fluorine fingerprint descriptors.

7.2 Calculation of fluorine fingerprints descriptors and ^{19}F CSA prediction by random-forest regression

Fluorine fingerprint descriptors were calculated following the method described by Vulpetti et al.[21]. The preparation of molecules and generation of fluorine fingerprints were performed using the open-source chem-informatics toolkit RDKit[22]. Topological torsion fingerprints were generated starting from the fluorine atom, with a maximum path length of 3, 4, 5, 6, 7 or 8. The choice of maximum path length affects the accuracy of the ^{19}F chemical shift prediction[21].

A random forest regression model[23] was fitted to the $\delta_\sigma^{\mathrm{eff}}$ values calculated with DFT methods using Gaussian 09[14] as described above. Fluorine fingerprint descriptors with varying maximum path lengths were used as features. To identify the optimal parameters for the random forest model, a grid search and 3-fold cross-validation was conducted, utilizing the relevant modules in Scikit-learn[24]. The optimal maximum path length and random forest parameters were determined separately for CF and CF_3 molecules (Table S5 and S9). The influence of the path length on predictor performance is shown in Figure S6 for CF_3 and Figure S7 for CF molecules.

Table S5 The performance of random-forest regression (RFR), given as the RMSE for $\delta_\sigma^{\text{eff}}$, is shown for different maximal path lengths, max(L). The corresponding model parameters for the random-forest regressor have been determined by grid search and 3-fold cross-validation. The RMSE has been calculated for 187 CF_3 containing molecules.

	CF_3					
	max(L)=3	max(L)=4	max(L)=5	max(L)=6	max(L)=7	max(L)=8
RMSE $\delta_\sigma^{\text{eff}}$ / ppm	2.0668	1.4247	0.8940	0.8601	0.9038	1.0728
max_depth	50	None	100	100	None	None
max_features	2	2	1	4	4	1
nr_estimators	100	100	100	200	200	100
min sample split	10	2	2	2	2	2

Table S6 The performance of random-forest regression (RFR), given as the RMSE for $\delta_\sigma^{\text{eff}}$, is shown for different maximal path lengths, max(L). The corresponding model parameters for the random-forest regressor have been determined by grid search and 3-fold cross-validation. The RMSE has been calculated for 324 CF containing molecules.

	CF					
	max(L)=3	max(L)=4	max(L)=5	max(L)=6	max(L)=7	max(L)=8
RMSE $\delta_\sigma^{\text{eff}}$ / ppm	9.29	7.58	6.76	3.02	2.26	2.15
max_depth	100	100	50	50	None	None
max_features	1	4	2	2	4	2
nr_estimators	100	100	200	100	100	200
min sample split	10	10	10	2	2	42

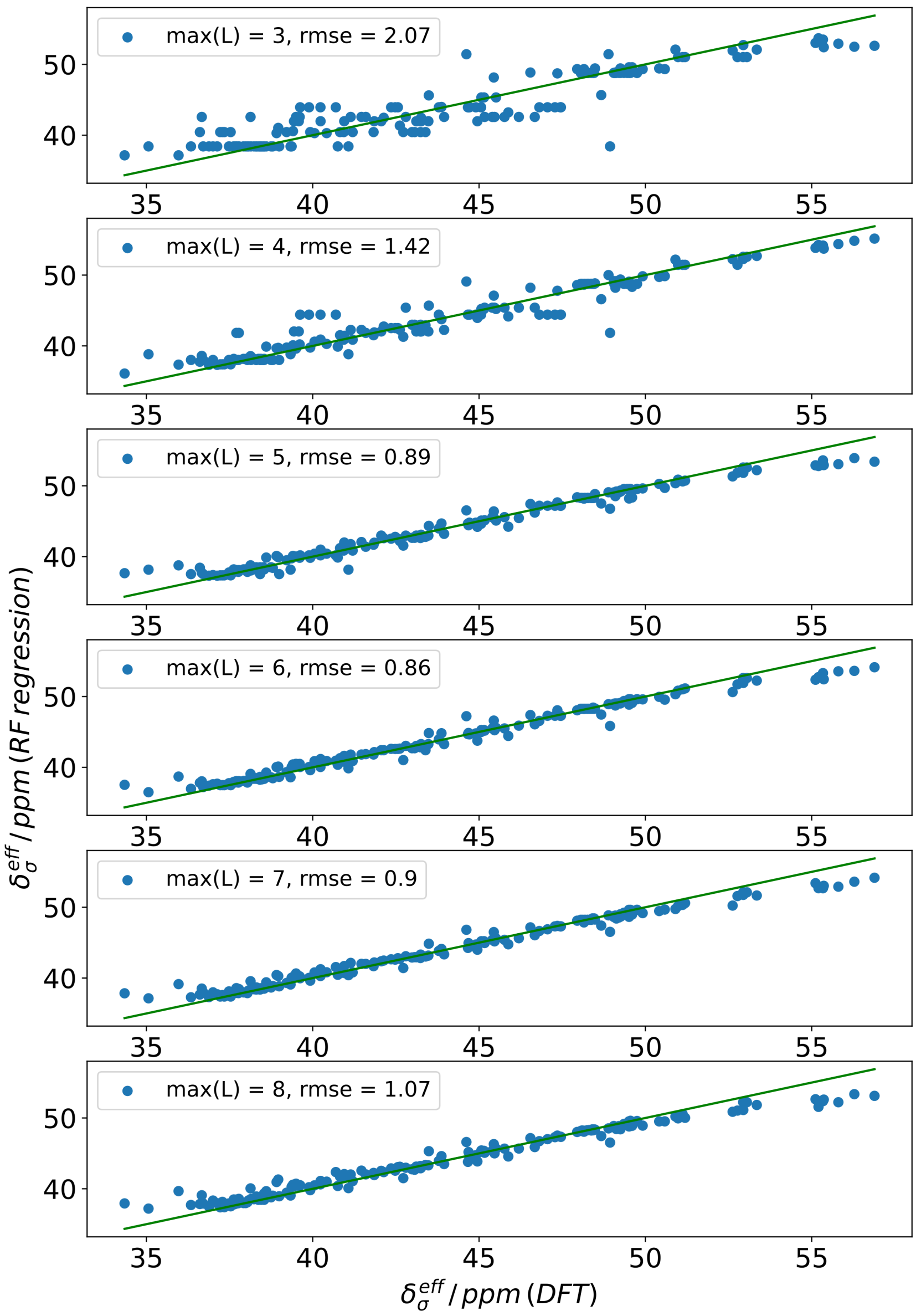

Fig. S6 Predicted versus calculated $\delta_\sigma^{\mathrm{eff}}$ values are show for 187 CF_3 containing molecules. These 187 molecules are a subset of the CF_3 screening library.

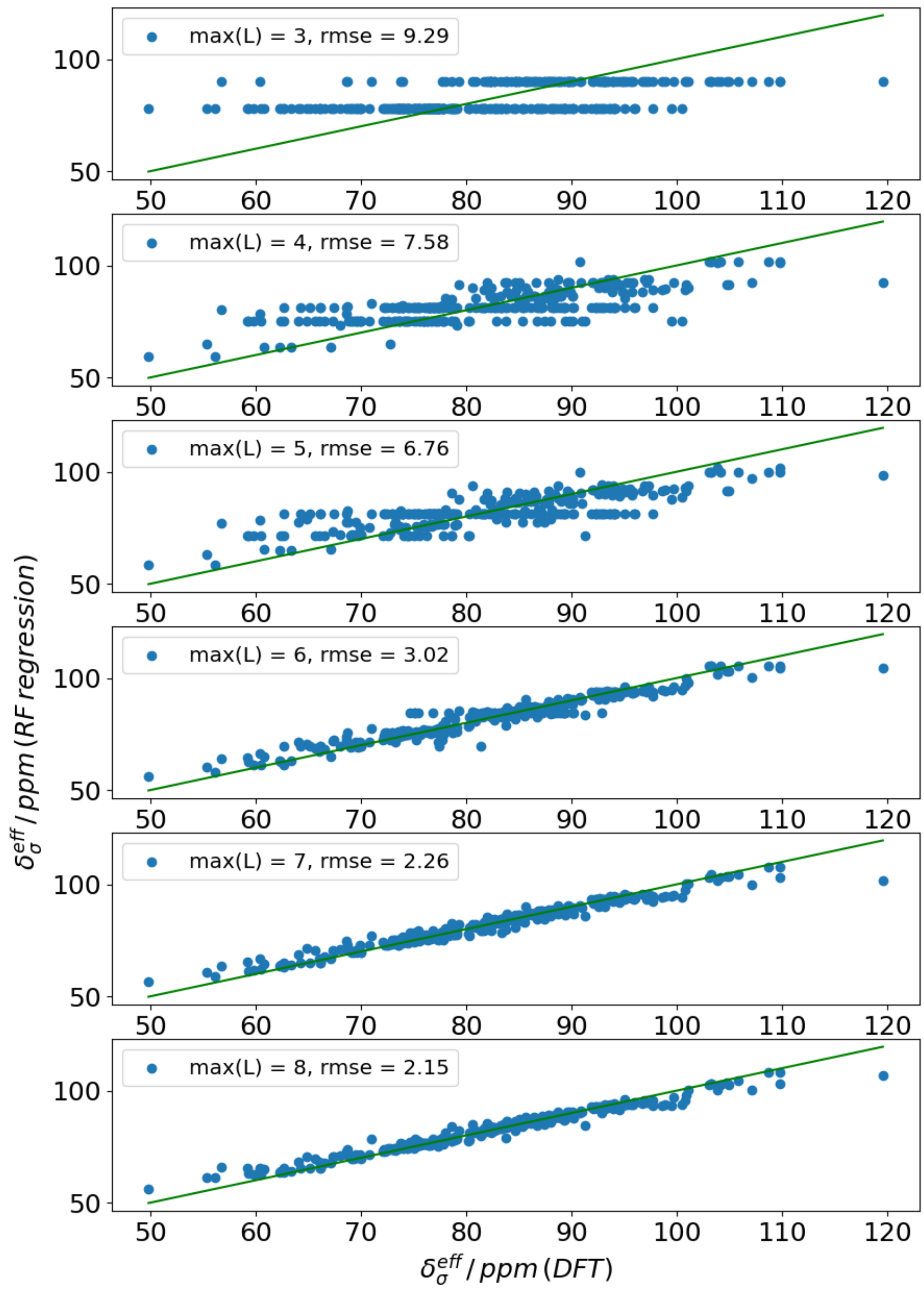

Fig. S7 Predicted versus calculated $\delta_\sigma^{\mathrm{eff}}$ values are shown for 324 CF containing molecules. These 324 molecules are a subset of the CF screening library.

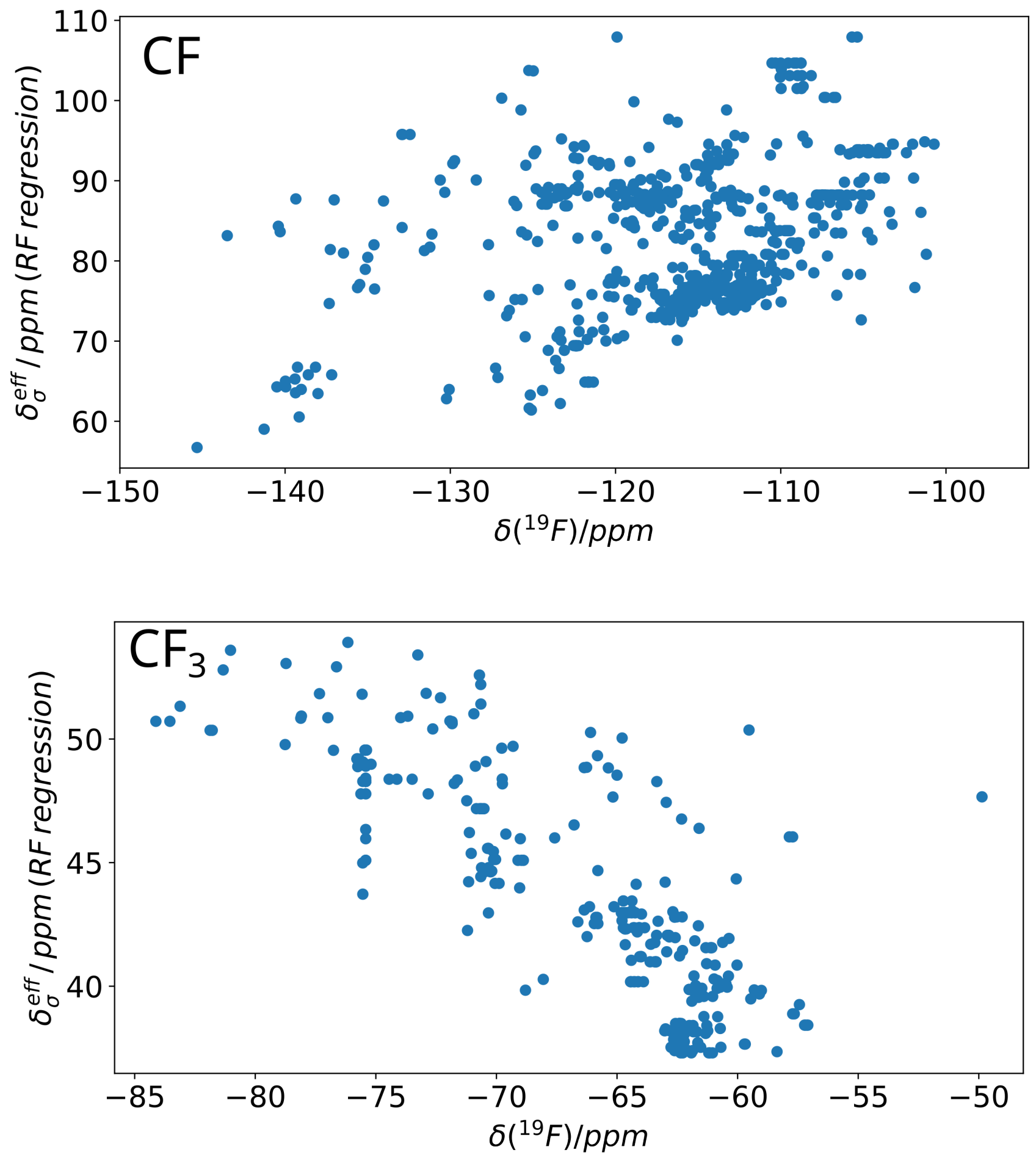

Fig. S8 The measured ^{19}F chemical shift is plotted against the effective reduced anisotropy, $\delta_\sigma^{\text{eff}}$, which was calculated using a random forest regression model for 674 CF and 361 CF_3 molecules. The results show only a weak correlation between the two parameters, indicating that the ^{19}F chemical shift is a poor predictor of $\delta_\sigma^{\text{eff}}$. The data are shown for all molecules which have been evaluated for purity, solubility and aggregation properties and also include molecules which are not included in the final screening set.

8 Comparison of CSA values predicted by machine-learning using random-forest regression with data from solid-state NMR

In order to demonstrate the universality of the machine-learning approach, we computed the CSA values using the described machine-learning random-forest (ML-RF) method for molecules (CF and CF_3) that are not included in the ^{19}F library OpenFL600 but for which CSA values have been reported from solid-state NMR measurements. The results, together with CSA values from DFT calculations are provided in Table S7 for CF_3 molecules.

Table S7 Comparison of DFT, ssNMR, and ML-RF CSA anisotropy values. Note that $\delta_\sigma^{\mathrm{eff}} = \delta_\sigma$ for CF_3 groups. $\delta_\sigma^{\mathrm{eff}}$ was calculated according to Equation S4. ssNMR data are from Grage *et al.*[25] and Dürr *et al.*[26], DFT values are from Rüdisser *et al.*[2].

**Molecule**	$\delta_\sigma^{\mathrm{eff}}$**(DFT)** / ppm	$\delta_\sigma^{\mathrm{eff}}$**(ssNMR)** / ppm	$\delta_\sigma^{\mathrm{eff}}$**(ML-RF)** / ppm
$4CF_3$-Phe	35.943	39	38.196
$3CF_3$-Phe	36.801	39	38.048
3,3,$3F_3$-Ala	45.549	44	48.467
5,5,$5F_3$-Leu	51.707	52	49.593
CF_3-BPG	53.203	52	50.154
4F-Phe		76 (L)	77.519
3F-Tyr		77 (D/L)	82.526

Table S8 SMILES codes for the molecules from Table S7.

**Molecule**	**SMILES**
$4CF_3$-Phe	[C@H]([NH3+])(Cc1ccc(cc1)C(F)(F)F)C(=O)O
$3CF_3$-Phe	[C@H]([NH3+])(Cc1cc(ccc1)C(F)(F)F)C(=O)O
3,3,$3F_3$-Ala	[C@H](C(=O)[O-])([NH3+])C(F)(F)F
5,5,$5F_3$-Leu	[C@@H](C[C@H](C)C(F)(F)F)(C(=O)[O-])[NH3+]
CF_3-BPG	[C@@H](C(=O)[O-])([NH3+])[C@]12C[C@](C1)(C2)C(F)(F)F
4F-Phe	C1=CC(=CC=C1C[C@@H](C(=O)O)N)F
3F-Tyr	C1=CC(=C(C=C1C[C@@H](C(=O)O)N)F)O

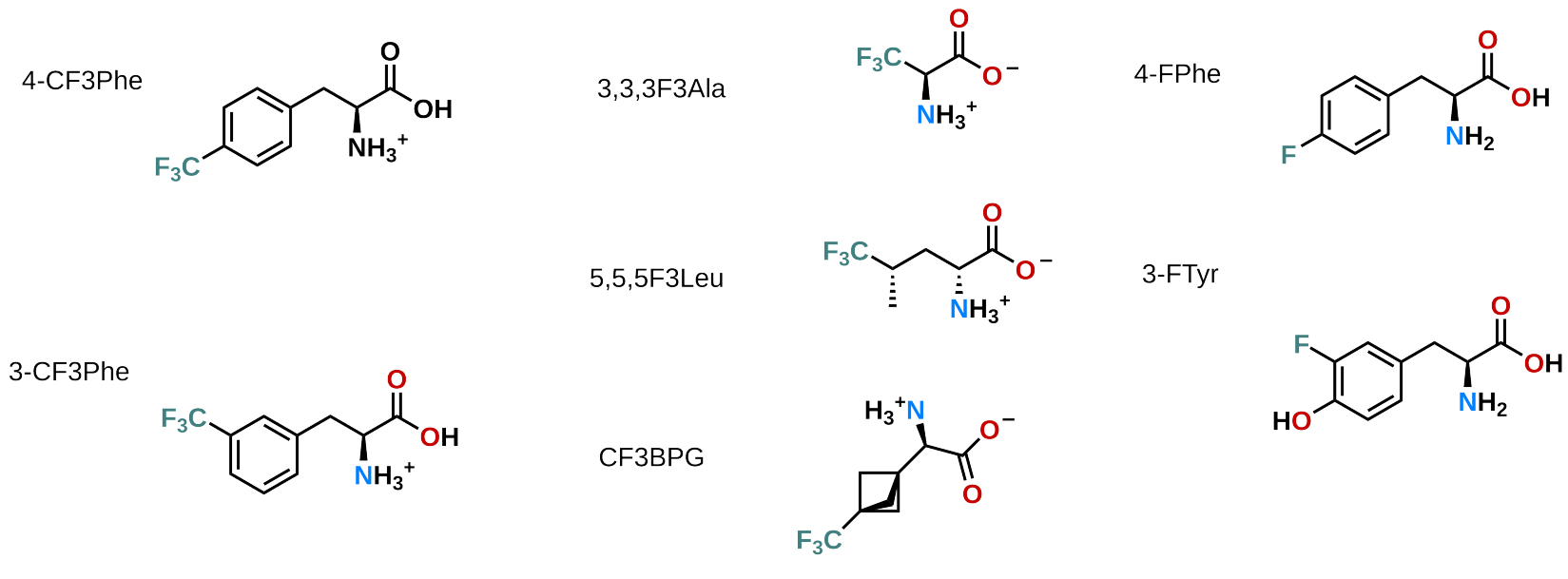

Fig. S9 Chemical structures of molecules for which ssNMR are available from Table S8.

Overall, the ssNMR and RF-derived CSA values show good agreement. For 4CF$_3$-Phe, the RF predictions are closer to the ssNMR data than the DFT results. We attribute this to the relatively large number of 4CF$_3$-phenyl motifs represented in the training set (OpenFL600 library). In contrast, for 3,3,3F$_3$-Ala, the DFT values are in better agreement with the ssNMR data than the ML-derived CSA values, likely reflecting the limited representation of structurally similar compounds in the training set. The DFT calculations performed in this study are of a high level of theory (M06 hybrid functional, AUG-cc-pVTZ basis). Nevertheless, their accuracy could be further improved by accounting for conformational variability. In the present work, CSA values were computed for the lowest-energy conformation only. For molecules that populate multiple conformations, a more rigorous approach would involve calculating CSA values for all relevant conformers and averaging them according to their populations. Such an analysis was not undertaken here, under the assumption that the lowest-energy conformation is the most populated in solution.

9 Computational Efficiency: Comparison of DFT and Machine Learning for ^{19}F CSA Calculation

The wall-clock time for calculating the ^{19}F CSA using DFT methods - including geometry optimization and frequency analysis to confirm the molecular conformation - was approximately 1.5 hours per molecule. These calculations were performed with *Gaussian09* on a cluster node equipped with a 64-core AMD© EPYC© 7H12 processors (2.6 GHz nominal, 3.3 GHz peak). In contrast, predicting the CSA parameters using our newly developed random forest regression model required less than one minute for a set of 300 molecules on a single core of a 12-core Intel© Core i7© i 14700 (2.1 GHz nominal, 5.4 GHz peak). This method eliminates the need for geometry optimization, as the calculation is based directly on the two-dimensional structure to generate the LEF fingerprints. The resulting speedup exceeds a factor of 7000 compared to the DFT-based approach.

10 ITC experiments for trypsin and TRY006

ITC experiments were performed on a PEAQ-ITC instrument (Microcal) with 12 injections of 3 µL every 150 s at 25°C following a first injection of 0.4 µL. Raw data were integrated, normalized for the molar concentration, and analyzed using Origin software, according to a 1:1 trypsin:TRY006 ratio binding model. Trypsin (Sigma Aldrich, T9201-100MG from bovine pancreas) solution in PBS buffer was freshly prepare to mitigate auto-proteolysis. A buffer exchange was performed using 10 kDa MWCO centrifuge concentrators (AMICON Ultra-4), concentrating 3x from 5 ml to 2 ml and adding 3 ml of PBS buffer, at 4000 rpm and 4°C. The concentration of trypsin was calculated from the absorbance at 280 nm ($A_{280\ nm} = 1$ corresponds to 1.54 mg / ml, MW = 24 kDa). TRY006 was dissolved in PBS buffer without any further buffer exchange.

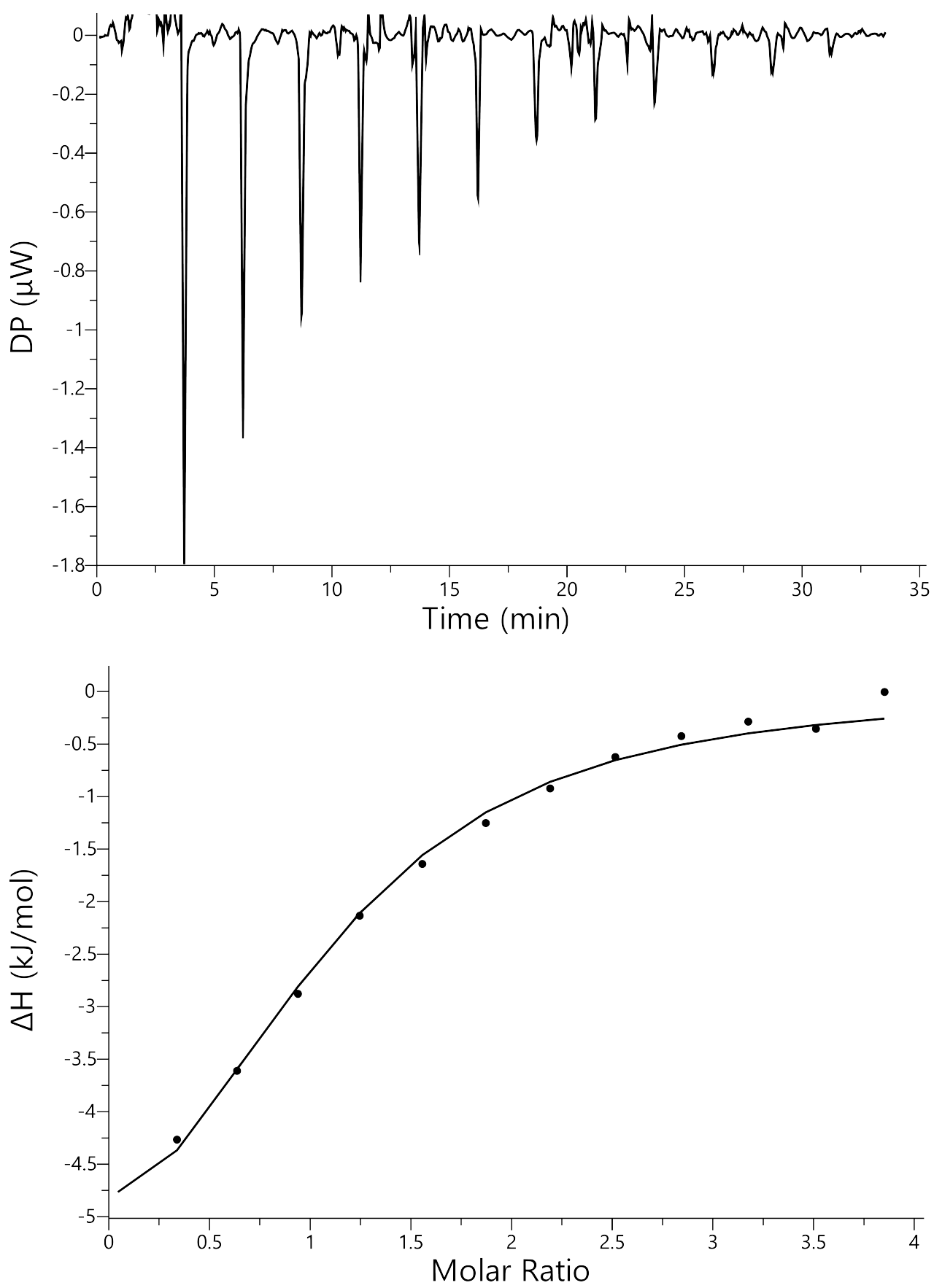

Fig. S10 ITC data for the titration of 50 µM trypsin with 1 mM TRY006 in PBS buffer, pH 7.4 (GIBCO 10010-015). A one binding-site model was fitted using the MicroCal software: K_D = (22.4 ± 2.31) µM, ΔH = (-6.98 ± 0.224) kJ/mol, ΔG = -26.6 kJ/mol, $-T\Delta S$ = -19.6 kJ/mol.

11 ITC experiments for trypsin and TRY001

Determination of the K_D of TRY001 using ITC under conditions identical to those used for the CSAKD experiments, *i. e.* in PBS buffer at pH 7.4, has been attempted. At conditions identical as those for TRY006 (50 µM Trypsin and 1 mM ligand), the exothermic effects were very weak. At higher concentrations of TRY001 of 10 mM, the exothermic effect increased, but the baseline did not stabilize at the same level as before the injections. Therefore, the ITC data for TRY001 were not suitable for fitting the K_D value. We note that a K_D of 86 µM has been determined by ITC at pH 8.0 in Tris buffer[27].

12 SMILES codes and ordering information of CSAKD ligands

Table S9 SMILES of ligands with dissociation constants (K_D^{CSA}) determined by the CSAKD method. The CSA anisotropies (δ_σ) and asymmetries η have been calculated using DFT methods.

receptor	ligand	SMILES	δ_σ, η / ppm	order nr.
PPAT	TFL355	`O=C(O)C1CCN(c2ccc3nnc(C(F)(F)F)n3n2)CC1`	42.8, 0	ENAMINE: EN300-25518
M^{pro}	TFL071	`O=c1[nH]c2cc(C(F)(F)F)ccc2n1C1CCNCC1`	36.4, 0	Otava: 5071628
	TRY013	`C1=CC2=C(C=C1C(F)(F)F)NN=C2N`	36.1, 0	Sigma: 5Z-0702
Abl1	ABL002	`CN(C)\C=N\C1=C(Cl)C=C(C=N1)C(F)(F)F`	36.6, 0	key organics: 8G-059
	ABL003	`FC(F)(F)C1=CC=C(NC(=O)CC#N)C=C1`	38.4, 0	key organics: BS-3667
trypsin	TRY001	`C1=CC(=CC=C1C(=N)N)C(F)(F)F`	36.7, 0	ENAMINE: EN300-73109
	TRY006	`C1=CC(=CC=C1C(=N)N)F`	88.3, 0.79	Sigma: CDS004472-100MG

References

(1) HAEBERLEN, U., *High Resolution Nmr in Solids Selective Averaging*; HAEBERLEN, U., Ed.; Academic Press: 1976, pp 1–190, DOI: https://doi.org/10.1016/B978-0-12-025561-0.50006-X.
(2) Rüdisser, S. H.; Goldberg, N.; Ebert, M.-O.; Kovacs, H.; Gossert, A. D. *Journal of Biomolecular NMR* **2020**, *74*, 579–594, DOI: 10.1007/s10858-020-00325-x.
(3) Hull, W. E.; Sykes, B. D. *J. Mol. Biol* **1975**, *98*, 121–153.
(4) Woessner, D. E. *The Journal of Chemical Physics* **1962**, *37*, 647–654, DOI: 10.1063/1.1701390.
(5) Goldman, M. *Journal of Magnetic Resonance* **2001**, *149*, 160–187, DOI: 10.1006/jmre.2000.2239.
(6) Lipari, G.; Szabo, A. *Journal of the American Chemical Society* **1982**, *104*, 4546–4559, DOI: 10.1021/ja00381a009.
(7) Lipari, G.; Szabo, A. *Journal of the American Chemical Society* **1982**, *104*, 4559–4570, DOI: 10.1021/ja00381a010.
(8) De La Torre, J. G.; Huertas, M. L.; Carrasco, B. *Journal of Magnetic Resonance* **2000**, *147*, 138–146, DOI: 10.1006/jmre.2000.2170.
(9) Skora, L.; Mestan, J.; Fabbro, D.; Jahnke, W.; Grzesiek, S. *Proceedings of the National Academy of Sciences* **2013**, *110*, DOI: 10.1073/pnas.1314712110.
(10) Skora, L.; Jahnke, W. *ACS Medicinal Chemistry Letters* **2017**, *8*, 632–635, DOI: 10.1021/acsmedchemlett.7b00084.
(11) Rößler, P.; Mathieu, D.; Gossert, A. D. *Angewandte Chemie International Edition* **2020**, *59*, 19329–19337, DOI: 10.1002/anie.202007715.
(12) Widmer, A. Witnotp: A computer program for molecular modeling. Basel, 1997.
(13) Clark, M.; Cramer, R. D.; Van Opdenbosch, N. *Journal of Computational Chemistry* **1989**, *10*, 982–1012, DOI: 10.1002/jcc.540100804.
(14) Frisch, M. J. et al. Gaussian˜16 {R}evision {C}.01, 2016.
(15) Rüdisser, S.; Vangrevelinghe, E.; Maibaum, J. In *Fragment-based Drug Discovery Lessons and Outlook*; Wiley-VCH Verlag GmbH & Co. KGaA: 2016, pp 447–486, DOI: 10.1002/9783527683604.ch19.
(16) Dunning Thom H., J. *The Journal of Chemical Physics* **1989**, *90*, 1007–1023, DOI: 10.1063/1.456153.
(17) Zhao, Y.; Truhlar, D. G. *Theoretical Chemistry Accounts* **2008**, *120*, 215–241, DOI: 10.1007/s00214-007-0310-x.
(18) Marenich, A. V.; Cramer, C. J.; Truhlar, D. G. *Journal of Physical Chemistry B* **2009**, *113*, 6378–6396, DOI: 10.1021/jp810292n.
(19) Ditchfield, R. *Molecular Physics* **1974**, *27*, 789–807, DOI: 10.1080/00268977400100711.
(20) Wolinski, K.; Hinton, J. F.; Pulay, P. *Journal of the American Chemical Society* **1990**, *112*, 8251–8260, DOI: 10.1021/ja00179a005.
(21) Vulpetti, A.; Landrum, G.; Rüdisser, S.; Erbel, P.; Dalvit, C. *Journal of Fluorine Chemistry* **2010**, *131*, 570–577, DOI: 10.1016/j.jfluchem.2009.12.024.
(22) RDKit: Open-source cheminformatics. https://www.rdkit.org/.
(23) Hastie, H.; Tibshirani, R.; Friedman, J., *The Elements of Statistical Learning: Data Mining, Inference, and Prediction*, 2nd; Springer US: 2016, p 767.
(24) Pedregosa, F.; Varoquaux, G.; Gramfort, A.; Michel, V.; Thirion, B.; Grisel, O.; Blondel, M.; Prettenhofer, P.; Weiss, R.; Dubourg, V.; Vanderplas, J.; Passos, A.; Cournapeau, D.; Brucher, M.; Perrot, M.; Duchesnay, E. *Journal of Machine Learning Research* **2011**, *12*, 2825–2830.
(25) Grage, S. L.; Dürr, U. H.; Afonin, S.; Mikhailiuk, P. K.; Komarov, I. V.; Ulrich, A. S. *Journal of Magnetic Resonance* **2008**, *191*, 16–23, DOI: 10.1016/j.jmr.2007.11.016.
(26) Dürr, U. H.; Grage, S. L.; Witter, R.; Ulrich, A. S. *Journal of Magnetic Resonance* **2008**, *191*, 7–15, DOI: 10.1016/j.jmr.2007.11.017.

(27) Lee, Y.; Zeng, H.; Ruedisser, S.; Gossert, A. D.; Hilty, C. *Journal of the American Chemical Society* **2012**, *134*, 17448–17451, DOI: `10.1021/ja308437h`.
